# A family-based study of genetic and epigenetic effects across multiple neurocognitive, motor, social-cognitive and social-behavioral functions

**DOI:** 10.1186/s12993-022-00198-0

**Published:** 2022-12-01

**Authors:** Ron Nudel, Richard Zetterberg, Nicoline Hemager, Camilla A. J. Christiani, Jessica Ohland, Birgitte K. Burton, Aja N. Greve, Katrine S. Spang, Ditte Ellersgaard, Ditte L. Gantriis, Jonas Bybjerg-Grauholm, Kerstin J. Plessen, Jens Richardt M. Jepsen, Anne A. E. Thorup, Thomas Werge, Ole Mors, Merete Nordentoft

**Affiliations:** 1 CORE - Copenhagen Research Centre for Mental Health, Mental Health Centre Copenhagen, Copenhagen University Hospital, Copenhagen, Denmark; 2 iPSYCH, The Lundbeck Foundation Initiative for Integrative Psychiatric Research, Aarhus, Denmark; 3 Institute of Biological Psychiatry, Mental Health Centre Sct. Hans, Mental Health Services Copenhagen, Roskilde, Denmark; 4 Mental Health Centre for Child and Adolescent Psychiatry - Research unit, Mental Health Services in the Capital Region of Denmark, Copenhagen, Denmark; 5 Department of Clinical Medicine, Faculty of Health and Medical Sciences, University of Copenhagen, Copenhagen, Denmark; 6 Psychosis Research Unit, Aarhus University Hospital - Psychiatry, Aarhus, Denmark; 7 Center for Neonatal Screening, Department for Congenital Disorders, Statens Serum Institut, Copenhagen, Denmark; 8 Division of Child and Adolescent Psychiatry, Department of Psychiatry, Hospital University Lausanne, Lausanne University, Lausanne, Switzerland; 9 Center for Neuropsychiatric Schizophrenia Research and Center for Clinical Intervention and Neuropsychiatric Schizophrenia Research, Mental Health Services in the Capital Region of Denmark, Copenhagen, Denmark

**Keywords:** GWAS, Neurodevelopment, Cognitive functions, Endophenotype, Parent-of-origin effect

## Abstract

Many psychiatric and neurodevelopmental disorders are known to be heritable, but studies trying to elucidate the genetic architecture of such traits often lag behind studies of somatic traits and diseases. The reasons as to why relatively few genome-wide significant associations have been reported for such traits have to do with the sample sizes needed for the detection of small effects, the difficulty in defining and characterizing the phenotypes, partially due to overlaps in affected underlying domains (which is especially true for cognitive phenotypes), and the complex genetic architectures of the phenotypes, which are not wholly captured in traditional case–control GWAS designs. We aimed to tackle the last two issues by performing GWASs of eight quantitative neurocognitive, motor, social-cognitive and social-behavioral traits, which may be considered endophenotypes for a variety of psychiatric and neurodevelopmental conditions, and for which we employed models capturing both general genetic association and parent-of-origin effects, in a family-based sample comprising 402 children and their parents (mostly family trios). We identified 48 genome-wide significant associations across several traits, of which 3 also survived our strict study-wide quality criteria. We additionally performed a functional annotation of implicated genes, as most of the 48 associations were with variants within protein-coding genes. In total, our study highlighted associations with five genes (*TGM3*, *CACNB4*, *ANKS1B, CSMD1* and *SYNE1*) associated with measures of working memory, processing speed and social behavior. Our results thus identify novel associations, including previously unreported parent-of-origin associations with relevant genes, and our top results illustrate new potential gene → endophenotype → disorder pathways.

**Supplementary Information:**

The online version contains supplementary material available at 10.1186/s12993-022-00198-0.

## Introduction

As a species, humans are adept at using communication (both verbal and nonverbal), mental facilities, social interaction abilities and fine motor skills in their everyday lives. These aptitudes mature during neurodevelopment. Some individuals, however, have non-typical neurodevelopment, which is associated with cognitive, motor, behavioral and/or social-cognitive impairments. Disorders characterized by these impairment are collectively known as neurodevelopmental disorders, and they often exhibit high comorbidity [1]. Many of these disorders have a strong genetic component, but they often exhibit both genetic and clinical heterogeneity [1–4]. Such a high degree of heterogeneity, in turn, encumbers studies into the molecular underpinnings of these disorders.

One strategy which has been proposed as a means to tackle this issue in psychiatric genetics is the use of endophenotypes. Endophenotypes are heritable traits that are (typically) convenient to measure and exhibit an association with the psychiatric condition; more formally, they are said to be heritable traits that are associated with the disease in the population, are primarily disease-state-independent and co-segregate with the disease in families (an additional criterion for complex diseases is that endophenotypes found in affected family members be found in non-affected family members at a higher rate than in the general population) [5]. Endophenotypes can also be quantitative, in which case they should be “milder” in unaffected relatives of affected individuals and correlated with the severity of the disease, and, if this correlation is not due to disease progression or medication, then it could suggest that the correlation with the disease is by way of disease liability [6]. Many traits that can be measured using standardized tests meet these criteria. Pertinent to this study is the case of heritable quantitative traits, which, in turn, may themselves be composites of different measures. For example, it has long been known that general intelligence is heritable [7]. Although the issue of what the intelligence quotient (IQ) itself measures is debated, as are the assumptions about the models estimating its heritability, the overall evidence from twin studies and other family-based studies suggests that a large proportion of the variation in IQ between individuals is due to additive genetic effects [8, 9]. Specifically, indices from subtests of the Wechsler Intelligence Scale for Children also have moderate to high heritabilities [10]. Moreover, specific tests designed to measure various phenotypic expressions of autism spectrum disorder, namely the “Strange Stories” test, which can identify Theory of Mind impairments, and the Social Responsiveness Scale, which provides a quantitative measure of autistic behavioral traits, have both been shown to have modest (“Strange Stories”) to high (Social Responsiveness Scale) heritabilities [11, 12]. In fact, measures from the Social Responsiveness Scale and from the Wechsler Intelligence Scale for Children have been successfully used as endophenotypes in studies of autism spectrum disorder (ASD) and attention deficit/hyperactivity disorder (ADHD) [13, 14]. Lastly, both motor skill and motor learning are also heritable [15, 16], and motor deficits have been suggested as an endophenotype for schizophrenia [17]. In psychiatry in general, endophenotypes tend to be electrophysiological e.g., electroencephalogram (EEG), eye tracking or certain reflexes [18] or behavioral e.g. gaze direction towards specific facial features [19]. An example of a relatively highly studied gene → behavioral endophenotype → disease pathway is that of the Calcium Voltage-Gated Channel Subunit Alpha1 C (*CACNA1C*) gene, which is a known susceptibility gene for several psychiatric disorders including schizophrenia [20]. A recent study showed that deletions in that gene in mice led to behaviors associated with psychiatric disorders [21].

Even though many genetic studies of the aforementioned traits and disorders (and of complex traits and diseases, in general) have been conducted, these studies together have not identified enough associations to account for the heritabilities of the investigated traits or diseases, a problem known as “the missing heritability” [22]. As genome-wide association studies (GWAS) become larger, more associations are identified at the conventional genome-wide significance threshold. However, there are other reasons why some associations elude the GWAS design, even as sample sizes grow larger: for example, there may be phenotypic heterogeneity not only across individuals, but also in the sense that different studies may use different definitions for disorders, different ascertainment criteria and/or different assessment tools, and, at times, the studied phenotypes themselves might reflect several overlapping underlying abilities. From the genetic perspective, an important reason is that the common GWAS study design, i.e., using only unrelated individuals and modeling only specific types of effects, might not capture all the aspects of the genetic architecture of a trait [22, 23]. Pertinent to this study is the case of the epigenetic phenomenon (i.e., a heritable phenomenon not caused by changes in the DNA sequence itself) known as parent-of-origin effect (POE), whereby the effect of an allele is dependent on its parental origin. Family-based genetic studies, where both parental DNA and proband DNA are available, are ideal for studying these effects. POEs have been implicated in many studies of complex traits and diseases [24]. Studies have shown that, when these effects do operate but are not modeled, they can be missed in traditional GWAS designs [25, 26]. Moreover, the same allele may have opposite effects when inherited paternally vs. maternally [25, 26].

Genomic imprinting is the epigenetic mechanism considered the primary underlying cause of POEs [27]. Imprinted loci are loci at which the two parental alleles are not functionally equivalent (and one of them may even be silenced completely). One molecular mechanism that could lead to imprinting is methylation (the presence of a methyl group on the DNA nucleotide). Allele-specific methylation in differentially methylated regions (DMRs), or, in this context, imprinting control regions, can lead to differential gene expression depending on the parental origin of the allele [27]. Modification of histones (basic proteins around which DNA is wound to form nucleosomes, a compact package of DNA which makes it possible for the DNA to fit within the nucleus of the cell) can also result in altered gene expression; protein complexes that modify histones covalently can lead to repression of transcription [28]. POEs may also result from mechanisms other than genomic imprinting, for example, bias in transmission of specific types of genetic, such as trinucleotide expansions variation, depending on the sex of the parent [29]. Several disorders which involve genomic imprinting have strong behavioral and cognitive manifestations. Perhaps the most often-cited examples thereof are Prader-Willi syndrome and Angelman syndrome. The genes involved in both of these disorders map to chromosome 15q11q13, but different genes are involved in the two disorders, and they exhibit opposite POEs (paternal for Prader-Willi syndrome and maternal for Angelman syndrome); similarly, the cognitive and behavioral deficits differ between the two disorders [30]. Most cases of these disorders are caused by a deletion of the parentally expressed DNA, but some cases are the result of imprinting defects, leading to aberrant methylation patterns [31, 32]. In the case of complex neurodevelopmental disorders, some notable examples for which POEs have been reported include specific language impairment [33, 34], dyslexia [35] and autism spectrum disorder [36]. A study of 97 traits in mice, where the parent-of-origin of alleles could be determined, found that most of them exhibited POEs, to which a large component of their heritability was attributable. Moreover, the study showed that non-imprinted loci could also exhibit POEs through interaction with imprinted loci [37]. These examples illustrate the importance of considering POEs in studying behavioral and cognitive phenotypes.

Our study aimed to examine both general genetic association as well as parent-of-origin effects, in a deeply phenotyped family-based cohort, in which families were chosen based on the presence (in at least one parent) or absence (in both parents) of a diagnosis of schizophrenia or bipolar disorder, and in which DNA from parents and children was collected, as well as data on a wide array of quantitative neurocognitive, motor, social-cognitive and social-behavioral traits [38]. In prior studies which used this cohort, several of the investigated traits have been shown to differ significantly between children of parents with no diagnosis of schizophrenia or bipolar disorder and children who had at least one parent with a diagnosis of schizophrenia. These included processing speed and working memory [39], social responsiveness [40], and motor function [41]. Interestingly, these studies did not find similar differences between children of parents with no diagnosis of schizophrenia or bipolar disorder and children who had at least one parent with a diagnosis of bipolar disorder.

The main goal of our study is thus twofold: (i) to find specific genotype–phenotype associations for the quantitative phenotypes from across the aforementioned domains, and (ii) to model POEs in addition to general association to identify associations that would not be captured in case–control GWAS designs. While we do not set out to show that the investigated traits are endophenotypes for specific disorders [as mentioned earlier, some of them have already been used as endophenotypes in previous studies, and they (or similar traits measured by other tests) have been shown to be heritable)], they are all inherently relevant to neurodevelopment in their own right. Moreover, a recent article examining the history of the use of endophenotypes in psychiatry proposed to expand the definition to include transdiagnostic traits, which are not necessarily associated with only one disorder [42]. In this context, identifying genetic variants influencing neurodevelopmental traits is an important endeavor in its own right. To our knowledge, this is the first study which examined these four neurodevelopmental domains in the same cohort, incorporating both general GWAS models and POE models.

## Materials and methods

### Participants

The sample used in this study is part of the Danish High Risk and Resilience Study—VIA 7 (hereafter the VIA 7 study) [38]. The VIA 7 study recruited children aged 7 and their biological parents. Families were recruited from Danish registries on account of having at least one parent with a diagnosis of either schizophrenia spectrum psychosis or bipolar disorder (“high risk” families) or as control families, in which neither parent had schizophrenia or bipolar disorder; however, these disorder were not investigated directly in this study. Overall, of the 402 children with genetic data included in this study (after quality control), 244 come from high risk families (schizophrenia: 147; bipolar disorder: 97), and 158 come from control families. The sample size varies per marker per analysis, as the number of informative children depends on the availability of trait data, marker (genotype) data, and, in the parent-of-origin analyses, parental genotypes as well. We therefore specify the number of informative children (probands) for all significant results individually. Regarding parental data, only genetic data were used in the association tests. After the quality control described below, there were 261.117 trios, 88.0364 child-mother duos, 24.1713 child-father duos, 17.0366 children, 0.352642 parents, 0.173135 mothers and 0.0495053 fathers (as well as 37.9879 parents without children in the dataset), on average per marker, as counted with PREMIM [43], without taking siblings into account. These numbers add up to ~ 391 (not counting parents without children in the dataset), which is the number of independent children with genetic data in our sample (11 families included a sibling as well).

### Phenotypic data

We investigated eight traits derived from different tests selected from the comprehensive battery of the VIA 7 study: **MABC** (total score from the Danish version of Movement Assessment Battery for Children (Movement ABC-2), 2nd edition [44]. *N.B.:* the norm sample for the Danish version was from the UK, but it has cross-cultural validity [45]); **WISC Coding** [score (total number correct) from the Coding subtest of the Danish version of the Wechsler Intelligence Scale for Children, 4th edition (WISC-IV) [46]]; **WISC Symbol Search** [score (total number correct) from the Symbol Search subtest of the Danish version of the WISC-IV]; **SSR** (score from the Strange Stories—Revised [47], based on the total number of correct answers to 8 mentalizing questions translated into Danish); **SRS** (T-score from the Danish version of the Social Responsiveness Scale (SRS-2) [48], 2nd edition, completed by the child’s teacher); **WISC Arithmetic** [score (total correct responses) from the Arithmetic subtest of the Danish version of the WISC-IV]; **WISC Letter-Number Sequencing** [score (number of correct trials) from the Letter-Number Sequencing subtest of the Danish version of the WISC-IV]; **RIST Index** [index score from the Danish version of the Reynold’s Intellectual Screening Test (RIST) [49]].

**Fig. 1:**
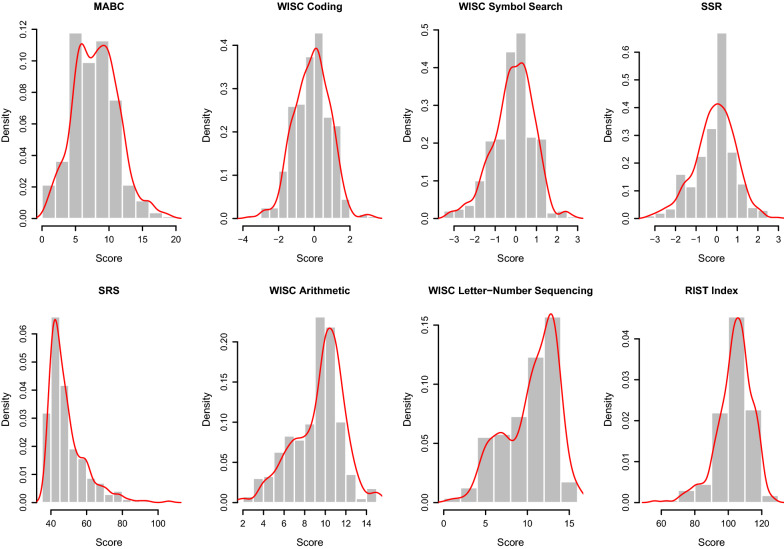
Histograms and density plots for all phenotypes across all children with non-missing phenotype values per trait. MABC, movement assessment battery for children; WISC Coding, coding subtest of the Wechsler Intelligence Scale for Children (WISC); WISC Symbol Search, symbol search subtest of the WISC; SSR, strange stories—revised; SRS, social responsiveness scale; WISC Arithmetic, arithmetic subtest of the WISC; WISC Letter-Number Sequencing, letter-number sequencing of the WISC; RIST Index, index score from the Reynold’s Intellectual Screening Test

**Fig. 2:**
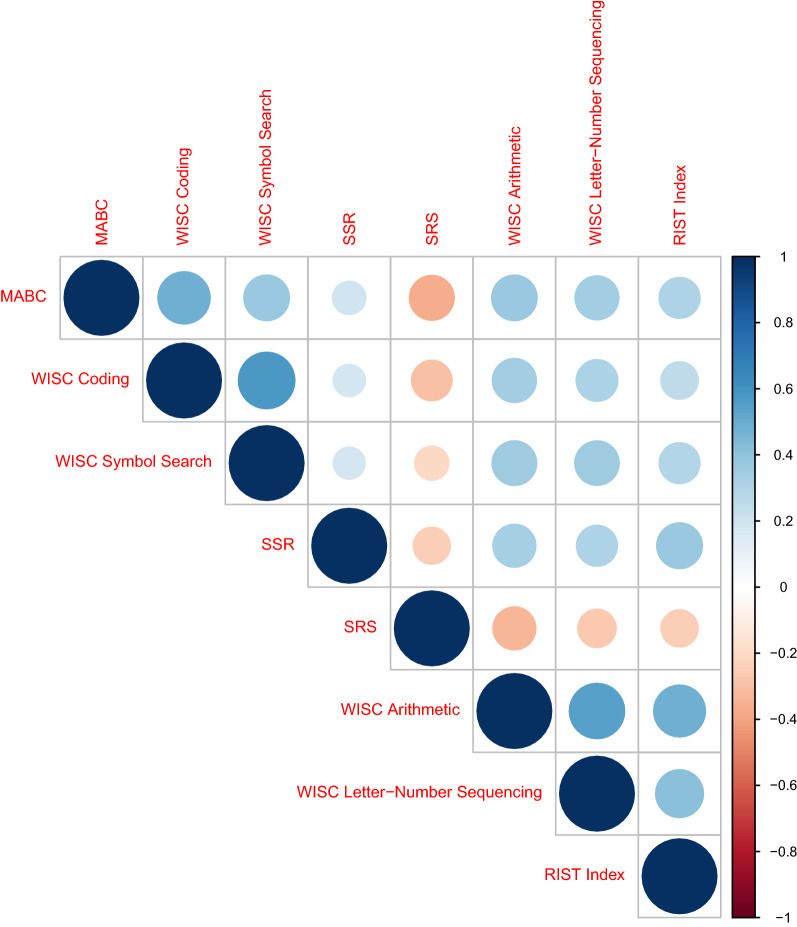
Pearson correlations across the investigated traits. All correlation coefficients were different from zero with P < 0.05

The WISC Arithmetic, WISC Letter-Number Sequencing, RIST Index and MABC scores were age-standardized based on the norms from the manual of each respective test. Where norms were not available for some tests or subtests e.g., when we used the versions of the WISC-IV Coding and Symbol Search subtests for children aged 8 to 16, or when there were no norms (SSR scores), the raw scores were rescaled into Z-scores in SPSS v25.0.0.2 using the mean of the population control subset of VIA 7 children, who were age-matched to the rest of the cohort. The SRS total T-score was not adjusted for age, as this score was not associated with age in children aged 7–15 [12]. More details about these tests can be found in previous publications on the VIA 7 study [39–41]. The distributions of the test scores for each trait are shown in Fig. 1, which contains histograms and density plots for the traits and was generated in R [50] v3.6.3 using the *hist* and *density* functions. We also calculated the pairwise Pearson’s correlation coefficients across the traits in the sample of children with genotypes used in this study, which are shown in Fig. 2. This was done using the Hmisc package v.4.7-0 [51] for R, and the plots were generated with the corrplot package v.0.92 for R [52]. Descriptive statistics for the traits are found in Table 1, which also includes p-values from the Shapiro–Wilk normality test as implemented in the *shapiro.test* function in R.

**Table 1:** Descriptive statistics for the investigated traits in the study sample. POE: parent-of-origin effect

Trait	Domain	The function measured by the test	Descriptive statistics for phenotypes in the full sample							General test^a^		Paternal POE test^b^		Maternal POE test^b^	
			Children with phenotype data	Mean	Median	Standard deviation	Minimum value	Maximum value	Shapiro–Wilk test p-value	Minimum Number of probands	Maximum Number of probands	Minimum Number of probands	Maximum Number of probands	Minimum Number of probands	Maximum Number of probands
MABC	Motor	A combined score from tests of manual dexterity, aiming and catching, and balance	399	8.105	8	3.31	1	19	0.0001	337	399	196	382	196	382
WISC Coding	Neurocognitive	Processing speed	401	− 0.134	− 0.059	0.992	− 3.61	3.219	0.0417	338	401	197	384	197	384
WISC Symbol Search	Neurocognitive	Processing speed	398	− 0.121	− 0.073	0.994	− 3.292	2.767	8.74 × 10^–5^	335	398	196	381	196	381
SSR	Social-cognitive	Theory of mind	400	− 0.1	0.046	0.999	− 3.227	2.909	1.69 × 10^–6^	337	400	197	383	197	383
SRS	Social-behavioral	Social responsiveness	345	49.014	46	10.441	37	106	4.68 × 10^–19^	292	345	172	331	172	331
WISC Arithmetic	Neurocognitive	Verbal working memory	398	9.475	10	2.396	2	15	1.18 × 10^–10^	335	398	196	381	196	381
WISC Letter-Number Sequencing	Neurocognitive	Verbal working memory	398	10.508	11	3.103	1	16	7.34 × 10^–13^	336	398	196	381	196	381
RIST Index	Neurocognitive	A combined score from tests of non-verbal intelligence (approximation of fluid intelligence) and verbal intelligence (a measure of crystallized intelligence)	401	103.85	105	10.458	56	127	5.75 × 10^–10^	338	401	198	384	198	384

^a^QTDT chooses informative probands from the pedigree file. Additionally, probands could have been excluded from the tests based on phenotype missingness, genotype missingness and/or problems with their IBD estimation with MERLIN

^b^In addition to the reasons for exclusion in the general test, probands could have also been excluded based on parental genotypes (see “Materials and methods”)

All traits deviated from normality to some degree. However, as the effective sample for the majority of our tests depends on the parental genotypes, it varies greatly across genetic markers (which are tested individually). Therefore, different subsets of children were used for different markers, and it is not practical to try to transform the scores so as to force them to have a normal distribution, when each transformation will not necessarily work for more than one marker. Moreover, transforming scores in this way would hinder the interpretation of the results, as the spaces between scores would have been changed unevenly, and, therefore, the interpretation of the effect sizes would be problematic. We discuss this at length in a previous paper, where we also examined the difference normalization had made for our top result [53]. Lastly, as we explain below, we used variance components (which assume normality) to correct for relatedness among the children within a given family. As we had only 11 families with more than one child, we investigated the effect of removing a child from each family and not modeling the variance components, and we saw that it had very little impact on our top result [53]; we therefore employ the phenotypic scores as detailed above, without an additional transformation.

### Genetic data

We had DNA samples from a subset of the VIA 7 study sample, and these were genotyped on the Illumina PsychChip v1-1_15073391_C, which had a loci count of 603,144 (according to the information lines in the Illumina manifest file for this array). The dataset has been described in detail in our previous studies [53, 54]. Briefly, the quality control (QC) steps for the samples and markers were as follows: initial QC on raw genetic data: individuals with low call rates or discordant sex information were removed in the first step, as were markers with a Gentrain score < 0.3. At this point 18 individuals had been removed (including one possible duplicate sample), and there were 600,282 markers left in the dataset. Subsequent QC was done with PLINK [55] v.1.90b5.2: individuals and markers with > 1% Mendelian errors were removed (N = 10). Genotypes with remaining Mendelian errors below this threshold were set to missing. Markers with > 5% missing data were removed (at this point all remaining individuals had < 5% missing data). Individuals with extreme heterozygosity rates (with a threshold of ± 3 SD from the sample mean) were removed (N = 21). Genetic ancestry was estimated in a principal component analysis (PCA). The threshold for the exclusion of samples was 2 SD above or below the VIA 7 mean for either PC1 or PC2, using the VIA 7 samples and the CEU, CHB, JPT and YRI HapMap samples to create the PC space, as described in a published QC protocol [56]. To reduce bias from population stratification, individuals of divergent ancestry were removed along with their relatives (N = 36), while the rest of the sample clustered with the CEU individuals. Individuals who exhibited cryptic relatedness or who were less related to biological family members than expected from pedigree information were removed (N = 13) (the Pi-hat threshold for the exclusion of individuals expected to be unrelated was 0.185). A Hardy–Weinberg Equilibrium (HWE) p-value threshold of 1 × 10^–6^ was employed for markers, as well as a minor allele frequency (MAF) threshold of 1% (in founders). Markers with a significant HWE p-value based on the above threshold or MAF below 1% were excluded. We removed one marker per pair in case of pairs of markers with identical positions included in the PsychChip, either using PLINK *--list-duplicate-vars suppress-first,* if the allele codes matched, otherwise prioritizing markers with rsIDs. The number of individuals removed during these steps was 64, including 3 duplicate samples (note that some samples were flagged in more than one step, in cases in which several checks were performed before the final exclusion of samples, namely, after the Mendelian errors check). In total, 1094 genotyped individuals and 299,604 autosomal markers passed these QC steps, from which we further removed 125 indels, for a total of 299,479 autosomal markers. Only this dataset of genotyped markers was used in this study. Positions in the text and tables are in genome build hg19. A minority of markers on the array had positions in the Illumina manifest file which differed slightly from the ones in dbSNP for the same major build. For that reason we checked all the probes from the manifest file for our array for the top hits in this study, as for the top hit in our previous study [53]. This was done using the UCSC Genome Browser BLAT tool, and, where a marker in our top hits had an rsID, we checked that the SNP was indeed 1 bp away from the probe. Otherwise we checked that the probe mapped to a position 1 bp away from the position in the manifest file. All probes mapped to a position 1 bp away from the position of interest (the direction depended on the strand), as expected. One marker among the top hits had an incorrect position in the manifest file, but the probe mapped to the right place.

### Statistical analyses—GWAS stage

In the GWAS stage, we used QTDT (quantitative transmission-disequilibrium test) [57] v2.6.1 for the statistical genetic analyses. MERLIN [58] v1.1.2 was used for estimating identity by descent (IBD) scores for each marker to be used by QTDT. Three tests were performed for each trait-marker combination: a general (i.e., not a POE test) total test of association using all family data (*qtdt -at*), a paternal parent-of-origin total test of association, in which only paternally inherited alleles were used (*-at -op*) and a maternal parent-of-origin total test of association, in which only maternally inherited alleles were used (*-at -om*). The total association model (as opposed to the orthogonal model, which QTDT can also run) is not a TDT, and it was used because it is more powerful in the absence of population stratification [59]. In this model, a combined between/within family component X, or Xpat and Xmat in the paternal and maternal tests, respectively, denoting the between/within effect on the means, is tested. X is the effect size reported for the QTDT analyses in this paper. X is estimated from the data in the full model and is fixed to zero in the null model. The likelihoods of these two models are then assessed through a likelihood ratio test, resulting in a χ^2^ statistic, which can be used to compute a one-sided p-value from the χ^2^ distribution. The tests in this study had one additional free parameter in the full model as compared with the null model, and so the χ^2^ statistic was evaluated with 1 degree of freedom. We included variance components in both models (*-wega*), incorporating an environmental component, a polygenic component and an additive major locus component. This allowed for the use of families with multiple children, although only 11 families included a sibling. Age was taken into account in the scoring of the phenotypes, as explained earlier. For all traits, a covariate for sex was added to both the null model and the full model.^1^ The Manhattan plots and the QQ plots were generated with the “qqman” R scripts by Stephen Turner and Daniel Capurso (with the (major update) version from April 19, 2011 for the former type of plot and the version from June 10, 2013 for the latter, available from: https://github.com/stephenturner/qqman/bloqb/v0.0.0/qqman.r). Regional association plots were generated with LocusZoom [60], after converting marker IDs to rsIDs (where possible) using a key from the Illumina website. The QTDT output files were tabulated using an in-house program (included in the Additional file 1), but the statistics for the top hits in our study were also examined manually using the raw QTDT output, and they matched the output of the program.

### Statistical analyses—post hoc tests for paternal and maternal allelic transmission differences

When a POE is detected with one parent, it does not mean that the other parent’s transmissions are significantly different. It could be that a child effect is significant and appears as such also when looking at paternally inherited alleles or only at maternally inherited alleles separately. Therefore, it is necessary to test for a difference between these parental allele transmissions. This can be achieved by controlling for risk parameters other than the POE parameter by including them in both the null and the full models. QTDT does not allow a free choice of parameters in the null and the full models, but it incorporates a test for the difference in the effects between the paternal and maternal allelic transmissions (*qtdt -at -ot*). In this test, the null model has X, and the full model has both X and Xmat. It is not possible to include Xpat instead of Xmat in the full model (to test for a POE when a paternal POE is suspected); therefore, as a precaution, we tested both parameterizations for a known POE using a different program, EMIM [43], which allows to model both parental risk parameters (one at a time in this case) in addition to the child risk parameter, and saw that the overall likelihood of the full model was roughly the same in both cases.^2^ Thus, we used this test to filter out associations that are significant in the GWASs when testing paternally inherited alleles and maternally inherited alleles separately, but which do not show a significant difference from the other parent’s transmissions. Note, however, that these models do not test for the simple parental effect at the locus of interest or for the type of POE (if the POE is real), and, for that reason, we only use them to test for the presence of a POE and not for effect estimation; we always report the effect of the allele from a test in which only Xmat or Xpat are included without X in the full model. Lastly, it should be noted that a POE may be significant with both paternal and maternal transmissions separately and there may still be a significant difference between them, if the association trends are discordant across both parental transmissions (i.e., the same allele may increase the score significantly when inherited from the mother but decrease it significantly when inherited from the father, or vice versa).

### The power and effective sample size of a QTDT analysis

The power of a QTDT analysis depends on several factors, including: the marker allele frequencies, the effect size, the linkage disequilibrium between the marker and the quantitative trait locus, the number of child genotypes in the analysis and the parental genotypes. Studies which evaluated family-based association methods used simulations of models with the above parameters to estimate the power of those methods. For dichotomous traits, for example, 300 case-mother duos offered reasonable power for detection of child genetic effects [61], when the effects were R_1_ = 1.5 and R_2_ = 2.25 (see second footnote for an explanation of the parameters), the baseline risk was 0.1, the significance level was 0.05, and the risk allele frequency was 0.3. When strong POEs operate and are included in the model, some methods achieved power of ~ 90% with as few as 100 case-parents trios [62], with I_p_ = 2.5 or I_m_ = 2.5, a significance level of 0.05, a baseline risk of 0.05 or 0.01 and a risk allele frequency of 0.3 or 0.1, for 20% and 80% of the population, respectively. With regards to quantitative traits, as relevant to this study, we considered published reports of simulations estimating the power of various QTDT models. For example, in the original QTDT paper, assuming a maximum D’, h^2^ of 0.1, a risk allele frequency of 0.5, a significance level of 0.001 and including parental genotypes, a sample of 480 children (families with a sibship of 1 and parental genotypes available) resulted in a power estimate of 97.4% [57]. In another study, a power of 74% was achieved with a sample size of 200, h^2^ of 0.1, and a risk allele frequency of 0.3 [63]. We can translate the effects of an allele into proportion of variance explained (PVE) using the following formula, taken from the supplementary note of a previous study [64]:

$${\text{PVE}}\, = \,{{\left[ {{2}\, \times \,\beta^{{2}} \, \times \,{\text{MAF}}\, \times \,\left( {{1} - {\text{MAF}}} \right)} \right]} \mathord{\left/ {\vphantom {{\left[ {{2}\, \times \,\beta^{{2}} \, \times \,{\text{MAF}}\, \times \,\left( {{1} - {\text{MAF}}} \right)} \right]} {\left[ {{2}\, \times \,\beta^{{2}} \, \times \,{\text{MAF}}\, \times \,\left( {{1} - {\text{MAF}}} \right)\, + \,{\text{SE}}\left( \beta \right)^{{2}} \, \times \,{2}\, \times \,{\text{N}}\, \times \,{\text{MAF}}\, \times \,\left( {{1} - {\text{MAF}}} \right)} \right]}}} \right. \kern-\nulldelimiterspace} {\left[ {{2}\, \times \,\beta^{{2}} \, \times \,{\text{MAF}}\, \times \,\left( {{1} - {\text{MAF}}} \right)\, + \,{\text{SE}}\left( \beta \right)^{{2}} \, \times \,{2}\, \times \,{\text{N}}\, \times \,{\text{MAF}}\, \times \,\left( {{1} - {\text{MAF}}} \right)} \right]}},$$

where β is the effect size, SE(β) is the standard error of β, MAF is the minor allele frequency of the marker (we used the MAF in founders), and N is the sample size (we used the number of probands). We do this for the top results of our analyses. One further point needs to be taken into consideration with regards to the current set of analyses: the power estimates from the literature are for the QTDT orthogonal model. In the absence of population stratification (as is the case in our study), the total association model can be used, and, all other things being equal, this model has greater power than the orthogonal model [59].

Regarding the sample sizes in the various tests, for the general test, all children who had non-missing genotypes and IBD information for a given marker and non-missing phenotypes were used in the test for that marker. In the POE tests, two groups of children are included: (i) children whose both parents are genotyped and where one parent is homozygous, or whose mother and father have different genotypes (in addition, when paternal parent-of-origin effects are tested, the father must be heterozygous and, when maternal effects are tested, the mother must be heterozygous), and (ii) all children with at least one homozygous parent, even if the other parent has a missing genotype [65]. This may reduce the sample size based on parental genotypes, which is why we report the number of informative probands (probands who meet all the above criteria (for the general test, and, where applicable, the additional criteria for the POE tests) for each association in the top results.

### Statistical analyses—correction for multiple testing and quality measures for GWAS results

We employed the following strategy for correction for multiple testing in this study: in the GWASs, we present all the associations that met the following two criteria: (i) they pass the conventional genome-wide significance threshold (P ≤ 5 × 10^–8^), (ii) for POE associations, they have P ≤ 0.0008 in the test of difference between paternal and maternal alleles, which was calculated as the conventional threshold (0.05) Bonferroni-corrected for the number of post hoc tests for POE associations which met the first criterion (n = 63). We then prioritize associations that, in addition to meeting the above two criteria, also meet the following criteria: (iii) they have a p-value (in the GWAS) equal to or below the conventional significance threshold (0.05) Bonferroni-corrected for the actual number of tests performed across all GWASs (n = 299,479 × 24), i.e., P ≤ 7 × 10^–9^; and (iv) at least 30 children had the minor allele for the associated marker (*N.B.:* this is not the same as the number of informative probands for QTDT, but rather it means that at least 30 children in the sample had the minor allele for the marker in question; while this does not guarantee that a specific number of children in a given test had the allele (as this also depended on the factors explained earlier), it could highlight associations for which the effect size is less likely to be biased due to one of the alleles being relatively rare). Associations surviving all four criteria are discussed in more detail in the Results section. For these associations, we also repeated the relevant association test while adding a covariate for the high risk status (HRS) of the family (that is, a dummy variable (0/1) for whether the child is from a family with a parent with schizophrenia or bipolar disorder, or from a family in which neither parent has either of these diagnosis). Additionally, we used EMIM v3.22 [43], a program for multinomial family-based genetic association models, to test for association between the top results (Table 2) and the HRS as a binary outcome. We used a model for child trend analysis [61] in which the factor by which the risk of disease is multiplied when the child has two risk alleles is constrained to be the square of the risk from having one risk allele, or, using the aforementioned notation, R_2_ = R_1_^2^. In this analyses we used both case and control family subsets, but we did not use controls without parents, since EMIM does not distinguish between controls and individuals with an unknown disease status (which means that parents, who by definition have an unknown HRS, might be used as controls if the child does not have genetic data for a given marker). The p-values for this test are derived from the χ^2^ distribution with one degree of freedom (since only one risk parameter was freely estimated in the full model), and the test statistic comes from twice the difference in the log-likelihoods of a null model (in which the multiplicative risk parameter is fixed to 1) and a full model, in which it is estimated from the data.

**Table 2:** Top results from the GWASs

Test	Trait	Marker ID	Chromosome	Position	MAF	Gene (protein coding)	Effect allele	Other allele	Probands	Effect	SE	χ^2^	p-value
General	WISC Arithmetic	psy_rs7826548	8	2955158	0.4717	*CSMD1*	C	T	389	− 0.199	0.036	31.12	2 × 10^–8^
General	WISC Arithmetic	rs2554728	8	3842999	0.2731	*CSMD1*	T	C	396	− 0.155	0.028	30.47	3 × 10^–8^
General	WISC Arithmetic	rs11995240	8	3848025	0.1691	*CSMD1*	T	C	396	− 0.016	0.003	29.84	5 × 10^–8^
General	WISC Arithmetic	rs17068473	8	3854987	0.2854	*CSMD1*	G	T	396	0.183	0.033	30.72	3 × 10^–8^
General	WISC Arithmetic	rs4875262	8	3876265	0.3858	*CSMD1*	G	A	396	0.262	0.046	31.95	2 × 10^–8^
General	WISC Arithmetic	rs2740929	8	3879918	0.4877	*CSMD1*	C	T	396	− 0.191	0.034	31.03	3 × 10^–8^
General	WISC Arithmetic	rs2740878	8	3918118	0.3288	*CSMD1*	A	G	396	− 0.309	0.054	33.05	9 × 10^–9^
General	WISC Arithmetic	rs2552166	8	3920395	0.1734	*CSMD1*	G	A	396	− 0.326	0.057	32.16	1 × 10^–8^
General	WISC Arithmetic	rs6049002	20	296172	0.3721		C	T	398	0.132	0.024	30.56	3 × 10^–8^
General	**WISC Arithmetic**	**rs6117457**	**20**	**733963**	**0.2883**		**T**	**C**	**398**	**− 0.359**	**0.062**	**33.9**	**6 × 10**^**–9**^
General	WISC Arithmetic	psy_rs79359757	20	937853	0.07381		C	T	398	− 0.053	0.01	30.01	4 × 10^–8^
General	WISC Arithmetic	rs7261002	20	1002656	0.1835		G	A	398	0.366	0.064	32.9	1 × 10^–8^
General	WISC Arithmetic	rs6118727	20	1030235	0.4465		A	G	398	0.071	0.013	30.15	4 × 10^–8^
General	WISC Arithmetic	rs200896	20	1789409	0.4292		A	C	398	− 0.326	0.056	33.38	8 × 10^–9^
General	WISC Arithmetic	psy_rs200888	20	1796461	0.3931		G	T	396	0.138	0.025	30.49	3 × 10^–8^
General	WISC Arithmetic	psy_rs4813309	20	1880550	0.1331	*SIRPA*	C	T	397	− 0.444	0.078	32.7	1 × 10^–8^
General	WISC Arithmetic	psy_rs6035018	20	1882954	0.19	*SIRPA*	T	C	398	− 0.392	0.068	33.07	9 × 10^–9^
General	WISC Arithmetic	exm1519370	20	1896100	0.3994	*SIRPA*	C	T	388	− 0.128	0.023	30.41	4 × 10^–8^
General	WISC Arithmetic	psy_rs73069290	20	1904515	0.06936	*SIRPA*	G	T	398	0.068	0.012	30.03	4 × 10^–8^
General	WISC Arithmetic	rs6035139	20	1936275	0.06014		G	A	398	− 0.905	0.163	30.94	3 × 10^–8^
General	**WISC Arithmetic**	**rs214831**	**20**	**2321363**	**0.3967**	***TGM3***	**G**	**A**	**398**	**− 0.395**	**0.067**	**35.28**	**3 × 10**^**–9**^
General	WISC Arithmetic	rs6137776	20	2338454	0.2392		T	C	398	0.281	0.05	31.82	2 × 10^–8^
General	WISC Symbol Search	rs2673776	6	152522812	0.4429	*SYNE1*	C	A	396	0.163	0.029	31.84	2 × 10^–8^
General	WISC Symbol Search	exm2270431	6	152644111	0.4565	*SYNE1*	C	T	396	0.146	0.026	31.44	2 × 10^–8^
General	WISC Symbol Search	psy_rs9478324	6	152677815	0.05058	*SYNE1*	G	A	396	0.287	0.052	30.51	3 × 10^–8^
Maternal	SRS	rs1451197	2	2741446	0.4993		G	A	213	− 4.888	0.872	31.42	2 × 10^–8^
Maternal	SRS	rs2176347	2	45968233	0.4473	*PRKCE*	T	G	231	− 3.441	0.624	30.39	4 × 10^–8^
Maternal	**SRS**	**rs7604835**	**2**	**152881908**	**0.1315**	***CACNB4***	**G**	**A**	**287**	**− 11.482**	**1.927**	**35.51**	**3 × 10**^**–9**^
Maternal	SRS	psy_rs77672109	4	35576468	0.01662		C	A	304	− 58.859	10.144	33.67	7 × 10^–9^
Maternal	SRS	rs11934637	4	92981939	0.0289		T	G	304	− 34.434	5.853	34.61	4 × 10^–9^
Maternal	SRS	psy_rs146416593	5	60662093	0.01592	*ZSWIM6*	G	A	324	− 33.216	5.844	32.31	1 × 10^–8^
Maternal	SRS	psy_rs79166730	5	175229558	0.01951	*CPLX2*	G	T	321	− 37.303	5.817	41.13	1 × 10^–10^
Maternal	SRS	exm573219	6	116325108	0.01879	*FRK*	G	A	325	− 58.949	9.981	34.88	4 × 10^–9^
Maternal	SRS	psy_rs78989171	8	49645477	0.01301	*EFCAB1*	G	T	308	− 58.867	10.204	33.28	8 × 10^–9^
Maternal	SRS	exm754630	9	74319677	0.01806	*TMEM2*	T	C	322	− 26.113	4.085	40.87	2 × 10^–10^
Maternal	SRS	rs1681993	12	63338414	0.01084		G	A	324	− 58.96	10.14	33.81	6 × 10^–9^
Maternal	SRS	rs7956933	12	63345018	0.01085		A	G	323	− 58.964	10.151	33.74	6 × 10^–9^
Maternal	SRS	psy_rs10860381	12	99309750	0.01375	*ANKS1B*	G	A	305	− 58.851	10.206	33.25	8 × 10^–9^
Maternal	WISC Arithmetic	rs11784069	8	2119582	0.3736		T	G	271	0.341	0.061	31.1	2 × 10^–8^
Maternal	WISC Arithmetic	rs7261002	20	1002656	0.1835		G	A	314	0.661	0.116	32.68	1 × 10^–8^
Maternal	WISC Symbol Search	rs2256135	6	152464839	0.4566	*SYNE1*	G	A	262	0.264	0.047	31.95	2 × 10^–8^
Paternal	RIST index	exm693219	8	28929739	0.02746	*KIF13B*	G	A	371	42.012	7.191	34.13	5 × 10^–9^
Paternal	SRS	exm109001	1	156314440	0.01517	*TSACC*	T	G	320	− 42.257	7.231	34.15	5 × 10^–9^
Paternal	SRS	psy_rs191695175	8	122841477	0.01016		T	C	326	− 47.854	5.665	71.36	3 × 10^–17^
Paternal	SRS	rs16908233	11	21604897	0.01158		A	G	317	− 34.271	5.86	34.2	5 × 10^–9^
Paternal	SRS	psy_rs117476444	13	113442872	0.02168	*ATP11A*	G	A	320	− 33.185	5.847	32.21	1 × 10^–8^
Paternal	WISC Arithmetic	rs11784069	8	2119582	0.3736		T	G	271	− 0.273	0.049	30.71	3 × 10^–8^
Paternal	WISC Arithmetic	rs2740939	8	3872513	0.4899	*CSMD1*	C	A	253	− 0.244	0.044	30.73	3 × 10^–8^

Results meeting criteria (i) and (ii) (see “Materials and methods”) are shown. Results meeting criteria (iii) and (iv) are shown in boldface. MAF: minor allele frequency (in founders; does not necessarily correspond to the effect allele frequency); SE, standard error

QTDT does not output standard errors (SEs) for the estimates it computes. In order to obtain SEs for the observed effect in the top associations in our results we used the following approach: using the χ^2^ statistics from the QTDT output, we calculated the error as ${\text{SE}} = \sqrt {\left( {{{{\text{X}}^{2} } \mathord{\left/ {\vphantom {{{\text{X}}^{2} } {\chi^{2} }}} \right. \kern-\nulldelimiterspace} {\chi^{2} }}} \right)}$, where X is the effect size from QTDT. This is an approximation of the SE, because it is calculated from a Wald statistic, whereas QTDT uses a likelihood ratio test for two nested models which differ by the presence of the effect of the genetic variant, but these two methods are at least asymptotically equivalent [66]. Lastly, the genomic inflation factor was calculated for each GWAS in R using the χ^2^ statistics from the QTDT output directly (as QTDT rounds the p-values themselves in the output) as follows: the median of the observed χ^2^ distribution from each GWAS divided by *qchisq(0.5, 1)*.

### Functional annotation of variants and genes

For functional annotation of variants, we used the eQTLGen [67] portal and the GTEx V8 portal [68] for finding expression quantitative trait locus (eQTL) associations and PhenoScanner [69] for finding DNA methylation and histone modification associations for the associations meeting our four study-wide criteria for significance. For gene-level annotation we used VarElect [70], which ranks genes based on their association with free text keywords using the GeneCards [71] database.

## Results

Across all 24 GWASs, 88 associations achieved genome-wide significance (P ≤ 5 × 10^–8^), of which 25 were highlighted in the general test and the rest were highlighted in the POE tests. Additional file 2: Fig. S1 shows Manhattan plots for all 24 GWASs, and Additional file 3: Fig. S2 shows the corresponding QQ plots. Across all analyses, the genomic inflation factor ranged from 0.967 to 1.077 (with a mean value of 1.008 and a standard deviation of 0.024). Of the POE associations among the aforementioned 88 associations, only 23 were significant in the test of difference between paternal and maternal alleles after correction for multiple testing (Methods), and the rest were therefore excluded from downstream analyses. The 48 remaining associations are shown in Table 2.

**Fig. 3:**
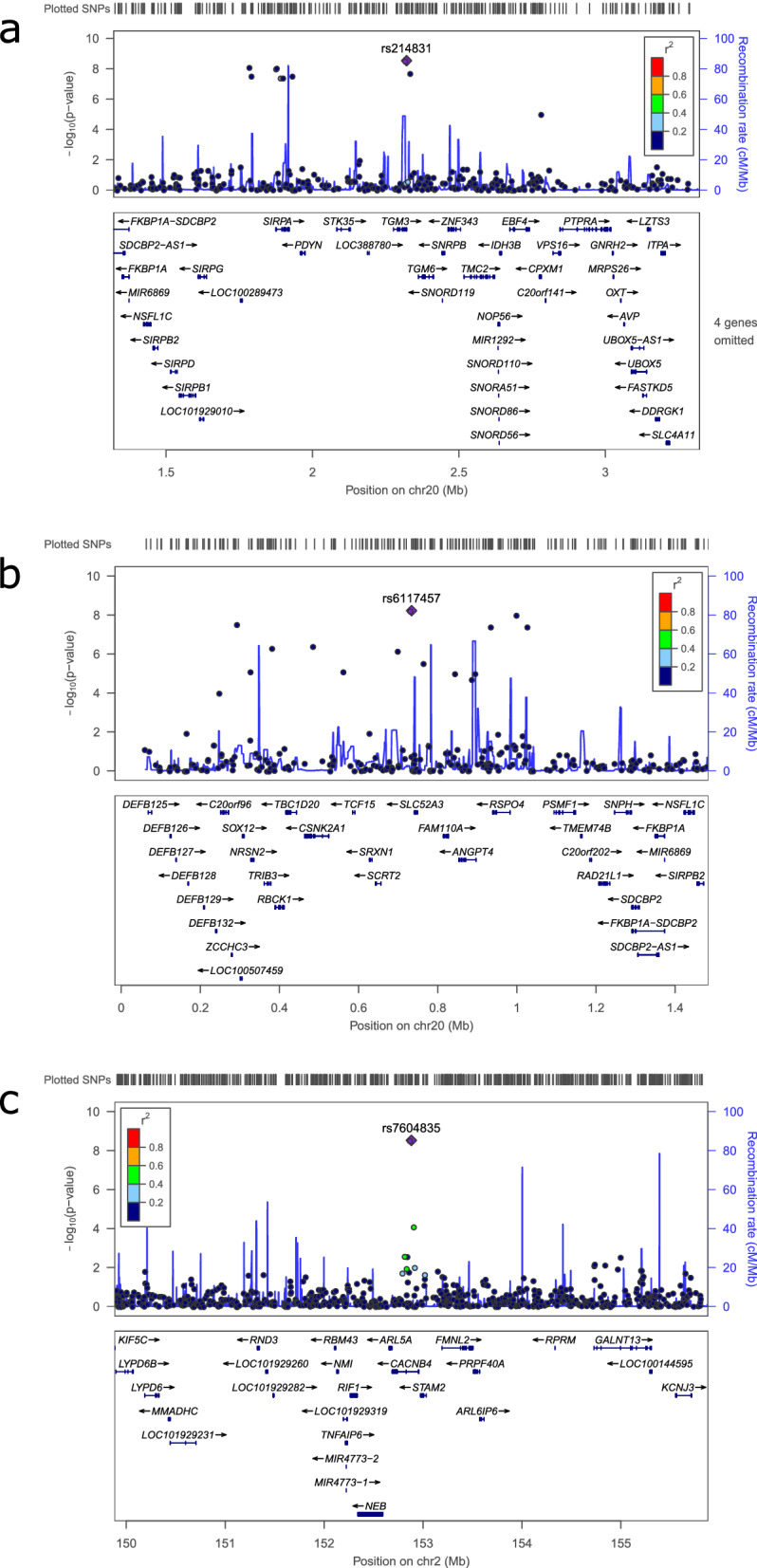
Regional association plots for associations surviving all four statistical quality criteria (Methods). **a** rs6117457 (general test, WISC Arithmetic); **b** rs6117457 (general test, WISC Arithmetic); **c** rs7604835 (maternal POE test, SRS)

Of the 48 associations that were genome-wide significant and, where applicable, showed a significant difference between paternal and maternal alleles, only 3 met our extra conditions pertaining to the study-wide significance level and a minimum number of 30 probands with the minor allele. Regional association plots for these 3 markers are shown in Fig. 3. We employed these extra criteria to identify more robust associations, especially because very rare alleles could lead to biased effect sizes. Of the 3 associations meeting all four criteria, 2 were with the WISC Arithmetic score and were highlighted in the general test and the remaining association was with the SRS score and showed a maternal POE. Two of these associations were with intragenic variants: rs214831 (general test, associated with WISC Arithmetic) in Transglutaminase 3 gene (*TGM3*) and rs7604835 (maternal POE test, associated with SRS) in the Calcium Channel, Voltage-Dependent, Beta 4 Subunit gene (*CACNB4*). Marker rs214831 was strongly associated with the expression of the gene it was located in, namely, *TGM3*, on eQTLGen (P = 5.72 × 10^–34^), whereby the A allele was associated with higher expression of the gene; in our study, the effect allele (G) was associated with a lower test score, suggesting that lower expression would be associated with a lower score. It was also associated with the expression of *PTPRA* in the basal ganglia on GTEx (P = 0.000022), with allele G being associated with lower expression. This marker remained at least nominally significant when adding a covariate for high risk status (i.e. for whether the child comes from a high risk family or a control family) to the model (P = 0.0312). Marker rs7604835, which showed a maternal POE in our study, was associated with multiple DNA methylation and histone modification sites on PhenoScanner (minimum P = 1.19 × 10^–45^), based on evidence from two different studies [72, 73]. This provides further support for the association with a POE at this locus. This marker remained genome-wide significant when adding a covariate for high risk status (P = 2 × 10^–9^). The last association which met all four criteria, namely, between rs6117457 and WISC Arithmetic in the general test, did not implicate any protein-coding gene, and we could not find any relevant prior association with it in the literature or functional databases. This marker did not remain significant when adding a covariate for high risk status (P = 0.0765). Translating the effects of the top markers into PVEs, we get: 0.078, 0.08 and 0.11 for rs6117457, rs214831 and rs7604835, respectively. The associations adjusted for HRS were in the same direction as before in all cases. It should be noted, however, that the interpretation of the models with the covariate for HRS can be difficult: both WISC Arithmetic and SRS are associated with the child’s schizophrenia family status in the VIA 7 study [39, 40]; since the covariate in this case may imply some genetic predisposition to schizophrenia, a disorder which is genetically correlated with cognitive traits [74], the same SNP could have some association with both the psychiatric disorder and the phenotype of interest. Furthermore, both schizophrenia and bipolar disorder are complex, meaning they have both genetic and environmental risk factors [75, 76]. Thus, the high risk status of the family, determined by the presence of a psychiatric diagnosis in one of the parents, is influenced both by genetic factors and environmental factors; the parental genetic factors influence both the child’s genetics (the exposure) and the high risk status of the family (the parent’s illness and potential covariate), which could influence the outcome in the child (the investigated trait). Similarly, environmental factors, which may be unmeasured (or external factors in general e.g., parental IQ), could influence both the high risk status of the family and the investigated trait in the child. In this scenario, adjusting for the covariate may reduce bias from possible confounding but introduce collider bias. A further complication would be the fact that most of our tests were for POEs, which limit the genetic causal path but not the causal path of the high risk status on which families were ascertained in this study. We therefore tested whether these markers were themselves associated with the high risk status as the outcome; none of the markers in Table 2 were associated with it after Bonferroni correction for multiple testing, and the top three markers were not nominally associated even before correction. Thus, if, for these markers, the high risk status of the family is not associated with the genetic exposure, then this eliminates both the potential confounding and potential collider bias from the model, even if HRS is not included as a covariate. Even though the high risk status refers to the parent and not the child’s phenotype, this lack of association could suggest that the traits highlighted in Table 2 might not be useful endophenotypes for schizophrenia or bipolar disorder, but might nonetheless be associated with other disorders.

Most of the associated markers in Table 2 (29 out of 48) fall within protein-coding genes. In total, 15 unique genes are implicated by at least one genome-wide significant association [meeting criteria (i) and, where relevant, (ii)] with a variant within them: *ANKS1B*, *ATP11A*, *CACNB4*, *CPLX2*, *CSMD1*, *EFCAB1*, *FRK*, *KIF13B*, *PRKCE*, *SIRPA*, *SYNE1*, *TGM3*, *TMEM2*, *TSACC* and *ZSWIM6*. Given that the associations in Table 2 were with the Arithmetic and Symbol Search subtests of the WISC, the SRS and the RIST, we used the following terms together with the gene names when running the VarElect analysis: autism OR "working memory" OR behavior OR communication OR intelligence OR "processing speed" OR "Wechsler Intelligence Scale for Children" OR "Reynolds Intellectual Screening Test" OR "Social Responsiveness Scale" OR schizophrenia OR "bipolar disorder". The last two terms were added because they represent the disorders based on which the VIA sample had been ascertained. Fourteen out of the fifteen genes were directly associated with at least one of the terms (i.e., the gene’s GeneCard contained the term), with the average number of associated terms per gene being 4.43 (± 2.41). Two genes were associated with 8 terms, the highest number of terms any one gene was associated with: Ankyrin Repeat and Sterile Alpha Motif Domain-containing Protein 1B (*ANKS1B*), and Synaptic Nuclear Envelope Protein 1 (*SYNE1*). Additional file 4: Table S1 lists all direct associations between the terms and the genes and a discussion of the scores. The gene with the highest VarElect score was CUB And Sushi Multiple Domains 1 (*CSMD1*), and the gene with the highest average disease causing likelihood was the aforementioned *CACNB4*.

## Discussion

Our study investigated eight neurocognitive, motor and social-cognitive and social-behavioral functions using a family-based GWAS design, including a general association test as well as tests of parent-of-origin effect tests. We have identified 48 genome-wide significant associations, of which 3 met our study-wide significance threshold. Our results highlighted several protein-coding genes, some of which have been implicated in prior genetic analyses of relevant phenotypes.

Two genes were highlighted through associations which met all four of our significance criteria: *TGM3* and *CACNB4*. The association with *TGM3* was further supported by the marker’s being an eQTL for the gene. This gene is involved in terminal epidermal differentiation and has been implicated in some cancers [77, 78]. In our study, the marker in this gene was associated with a measure of working memory. Interestingly, previous studies have found relevant associations between the gene and related phenotypes: a study of the RNA blood transcriptome of patients with Alzheimer’s disease (AD), a disease which involves severe memory impairments, found that the largest expression fold change among differentially expressed genes across AD cases and controls was with *TGM3* [79]. Genes of the same family have been implicated in several neurodegenerative diseases [80]. Also of note, the associated marker in our study was also a brain eQTL for *PTPRA*, a gene which is important for hippocampal neuronal migration; mice deficient for the PTPRA protein exhibit impairments in learning and short-term memory [81]. The association between social responsiveness (SRS) and *CACNB4* was with a maternal POE. This marker was also associated with methylation and histone modifications sites, providing further support for a POE. The gene encodes a member of the beta subunit family of voltage-dependent calcium channels, and it belongs to a family of genes which has been implicated in several psychiatric and neurodevelopmental disorders, including autism spectrum disorder, across many studies [82]. The subunit encoded by *CACNB4*, specifically, is highly expressed in the brain and is prominent in the cerebellum [83]. A recent study found that a pathogenic missense variant in this gene resulted in a severe neurodevelopmental impairment which included intellectual disability, language impairment, movement impairment and seizures [84]. When adding a covariate for high risk status to the statistical models for the top associations, we observe that it either slightly improved the association (with SRS, maternal POE test) or reduced it drastically (with WISC Arithmetic, general test). Whether or not it is appropriate to include this covariate in the model depends on the causal paths between the genetic variant, the trait, and the covariate, which are complex and not known. Hence, the interpretation of these post hoc tests should be done with caution.

Among the other genes in Table 2, three genes were highlighted in the functional annotation either as having the highest VarElect score or as being associated with the largest number of terms: *CSMD1*, *ANKS1B* and *SYNE1*. *CSMD1* is of particular interest because it has been implicated in schizophrenia [85–87]. Interestingly, in our study, this gene was implicated through markers associated with a measure of working memory; a study of this gene reported that a schizophrenia risk variant in *CSMD1* was associated with spatial working memory [88]. This could illustrate the effect of a genetic variant on an endophenotype for schizophrenia. In this context it is also important to note a proposal to redefine the notion of endophenotype in psychiatry to allow it to include transdiagnostic traits that may be shared across several disorders [42]. *ANKS1B* was implicated through the association between the marker psy_rs10860381 and social responsiveness in the maternal POE test. This gene encodes an activity dependent postsynaptic effector protein highly expressed in the brain, and it has been implicated in a wide array of neurodevelopmental phenotypes [89]. Importantly, haploinsufficiency of this gene in a mouse model resulted in impaired social interaction and sensorimotor dysfunction, which are core features of autism spectrum disorder [90]. Even more importantly, this gene exhibits allelic expression imbalance in the brain, which could be an outcome of genomic imprinting (which could result in a POE), although this is only one possible explanation [89]. *SYNE1* was implicated through the associations between several markers and processing speed (WISC Symbol Search) in the general test. The gene encodes a protein that is involved in anchoring specialized myonuclei underneath neuromuscular junctions, but it is also expressed in the brain—predominantly in the cerebellum [91]. It has been implicated in a recessive form of cerebellar ataxia, which may also include cognitive deficits [91]. Interestingly, individuals with *SYNE1* mutations exhibit processing speed deficits compared with controls [92], which is in line with our result showing association between this gene and processing speed in the general test. Both *SYNE1* and *TGM3* have been highlighted in a study of de novo mutations in autism spectrum disorder [93].

Some of the other associations in Table 2 are also of note. The paternal POE association between social responsiveness and rs191695175 was the most significant association in our study. The minor allele frequency for this marker was very low at ~ 0.01 (in founders), which could lead to a biased effect size. However, this marker is found on chromosome 8 in chromosomal band 8q24.13, a locus which was part of a suggestive linkage peak for the same trait, namely, SRS, in a genome-wide linkage study [13]. The same locus also showed linkage to SRS in addition to an anxiety score and a score for pragmatic language skills, in another study [94]. These studies, however, did not model POEs. Thus, even though we may not be able to trust the estimated effect size for this locus, the association itself might be valid and supported by previous studies, and it is possible that the POE, if it indeed operates at this locus, contributed to the stronger signal in this study as compared with previous studies. There have been other previous studies which included similar phenotypes, such as social interaction and social communication (neither was measured with the SRS), but they did not model POEs, and their significant results do not overlap with ours [95, 96]. We also observe an interesting association trend with marker rs11784069: allele T, when inherited from the mother, is associated with a higher WISC Arithmetic score (better working memory functions), but, when inherited from the father, it is associated with a lower score (Table 2). This is an illustration of the phenomenon mentioned in the introduction, namely, opposite POEs of different parental types at the same locus, which has been observed for other quantitative traits in humans. This marker is a highly significant eQTL for *MYOM2* on eQTLGen (P = 3.2717 × 10^–310^) and GTEx (P = 9.5 × 10^–20^) in whole blood. Interestingly, the mouse ortholog of this gene, *Myom2*, was significantly upregulated and had the fifth largest fold change among upregulated genes in the hippocampus of memory-enhanced mice in one study [97], which is relevant for the association in our study, as the WISC Arithmetic score is a measure of working memory.

### The top results in the context of endophenotypes and the investigated domains

The traits implicated by the top results in our study, namely, SRS (social responsiveness) and WISC Arithmetic (working memory), had been proposed as endophenotypes for ASD and ADHD, respectively [13, 98–100]. However, these studies did not identify links between specific genes and these endophenotypes at a genome-wide significant level; they focused on linkage analyses or candidate genes, and, where association was modeled, it was only suggestive. Thus, our study provides genetic evidence for the missing piece in the pathway from gene to disorder through endophenotype, namely: *TGM3* → working memory → ADHD and *CACNB4* → social responsiveness → ASD, through the top genetic associations we identified. Similarly, memory impairments, including verbal working memory impairment, are common feature of schizophrenia [101], suggesting further pathways between *TGM3*, *CSMD1* and *PTPRA* and schizophrenia through the working memory endophenotype. The highlighted associations in Table 2 belong to the neurocognitive and social-behavioral domains. This does not mean that traits from the other domains would not make good endophenotypes; our study did not examine that, and the lack of genetic association could result from lower heritability for those traits and/or insufficient sample sizes.

### Limitations of our study

Our results should be evaluated in the light of several potential limitations. Firstly, our study sample was a family-based sample, and, as such, not a very large one. While this has the advantage of our being able to have a deeply phenotyped sample, it can be detrimental to genetic association studies. While, as shown in previous simulations studies of QTDT models, our sample should be large enough to detect some effects, it is to expected that only strong effects could be detected in our sample, which can explain why the majority of our genome-wide significant associations were intragenic. It should also be emphasized that some of the effect sizes could be overestimated due to confounding. As it is difficult to determine the appropriateness of the adjustment for high risk status, it should be borne in mind that the effects for some associations might not be accurate. However, since the GWASs were performed with the goal of discovering new genetic associations for downstream analyses and not for estimating their effects, we adopted this approach rather than potentially over-adjust the models, as discussed earlier. Another limitation is that we did not have a suitable replication sample which included the same phenotypes and genetic data from children and parents. Although our candidate genes have been highlighted in previous studies of related traits, providing more credibility to their association with our traits, the associations with specific variants need to be replicated in an independent sample.

### Future perspectives

It has been shown that the heritability of cognitive ability increases from childhood to young adulthood [102]. Interestingly, a similar trend (reaching its peak around age of 13 for girls and 14 for boys) was observed for height [103]. When the proportion of phenotypic variance explained by genetics increases, the proportion of the variance explained by the environment decreases, and vice versa. In the case of height, this trend could reflect the effect of early childhood living conditions and/or prenatal environmental factors [103]. For cognitive ability, the authors theorize that this trend could be a result of genotype-environment correlation, whereby their genetics influences children increasingly in selecting, modifying and creating their own experiences as they grow up [102]. From the statistical genetic perspective, a higher heritability means that more genetic associations could be identified if the sample of children were studied when they are older; this could mean that repeating the analyses within the VIA sample with these functions measured in early adulthood could result in further associations. Furthermore, functional studies of the genes highlighted in our study could provide further insight into the molecular etiologies of the neurodevelopmental disorders whose endophenotypes were investigated in this study.

## Conclusions

Our study identified several candidate genes for social-behavioral and neurocognitive functions, implicated either through a general test, or a test of POEs; associations in the latter test were also supported by external studies which had identified methylation or histone modification sites associated with the relevant marker. Importantly, most of our genome-wide significant associations were within protein-coding genes, and many of these had previously been implicated in studies of related traits and disorders, although many of these previous associations were with rare and/or deleterious mutations. Our study provides further evidence to the effect that common variants may influence related traits in individuals not diagnosed with severe mental disorders, and it further supports a role for the highlighted genes in the studied traits, which can be seen as a replication of those genes’ implications in the previous studies. We did not identify significant associations for traits in some of the other functions/domains included in this study; this could be the result of the lower heritability of those traits, as well as potentially smaller effects that could not be discovered in the VIA sample. Our results also illustrate the usefulness of modeling POEs in human genetic studies, and, while previous studies focused on an array of quantitative non-social-cognitive, non-social-behavioral, and non-neurocognitive traits, our study highlights the presence of potential POEs in several of these traits studied in a systematic way, thus providing further evidence for this phenomenon in humans.

## Supplementary Information

**Additional file 1.** Archive file containing std_qtdt, which can parse and tabulate the QTDT output.

**Additional file 2: Figure S1.** Manhattan plots for all 24 GWASs.

**Additional file 3: Figure S2.** QQ plots for all 24 GWASs.

**Additional file 4: Table S1.** Direct associations from VarElect for genes in Table 1.

## Data Availability

Access to the dataset used in the current study is available from the corresponding author upon reasonable request. The program used to tabulate the QTDT output is available in a additional file accompanying this article.

## Footnotes

1 *N.B.:* The Danish manual for the SRS we used did not include a sex-adjustment for the T-score; hence, we used a covariate for sex for this trait as well.

2 Consider a previously reported association [34, 54] with a paternal POE for specific language impairment: rs4280164 allele A having a paternal imprinting parameter I_p_ (a multiplicative factor by which the probability of disease is multiplied if the child receives a (paternal) copy of the effect allele from their father) = 0.255 with P = 2.918 × 10^–8^ (1 degree of freedom). In this case, the null model has all risk parameters fixed to 1, and in the full model I_p_ is freely estimated; the two models are assessed using a likelihood ratio test. As the EMIM software (43) (used in the original study) allows many parameterizations, it is also possible to include a free R_1_ parameter (the factor by which the disease risk is multiplied if the child has a single copy of the effect allele, and assuming that the risk from the child’s having two risk alleles is R_2_ = R_1_^2^) in both the null and full models and a free I_p_ parameter only in the full model. This results in a χ^2^ of 28.752840302, P = 8.22 × 10^–8^ (1 degree of freedom), suggesting that the paternal POE is still significant even when allowing for a child effect. Importantly, even when estimating the maternal imprinting parameter, I_m_ (a multiplicative factor by which the probability of disease is multiplied if the child receives a (maternal) copy of the effect allele from their mother), instead of I_p_, at the same locus (with R_1_), the likelihood of the full model here provides very similar evidence of association (χ^2^ of 28.752840295436), although the parameter estimates are different. Similarly, even if we do not make the assumption that R_2_ = R_1_^2^ (i.e., we freely estimate both R_1_ and R_2_ in the null and full models), we still obtain very similar likelihoods, resulting in χ^2^ = 25.88917562832 when estimating I_p_ in the full model, and χ^2^ = 25.889147279256 when estimating I_m_ in the full model (in both cases including free R_1_ and R_2_ in both the null and full models), both obtaining P = 3.62 × 10^–7^ (1 degree of freedom). We believe that QTDT is doing something similar with its test of allelic difference, and that a test of either combination can be used to determine the presence of a POE, even though QTDT always models the maternal POE effect parameter as the additional one.
