# Supplementary figures and images for: A family-based study of genetic and epigenetic effects across multiple neurocognitive, motor, social-cognitive and social-behavioral functions
Source: Behav Brain Funct. 2022 Dec 1;18:14. doi: 10.1186/s12993-022-00198-0 (PMC9714039; doi:10.1186/s12993-022-00198-0)

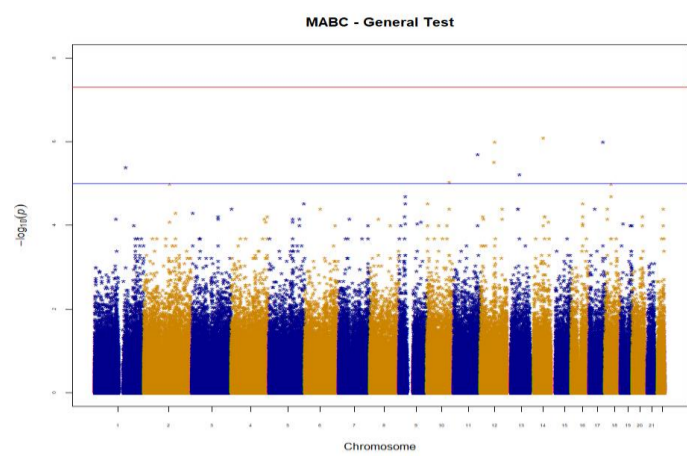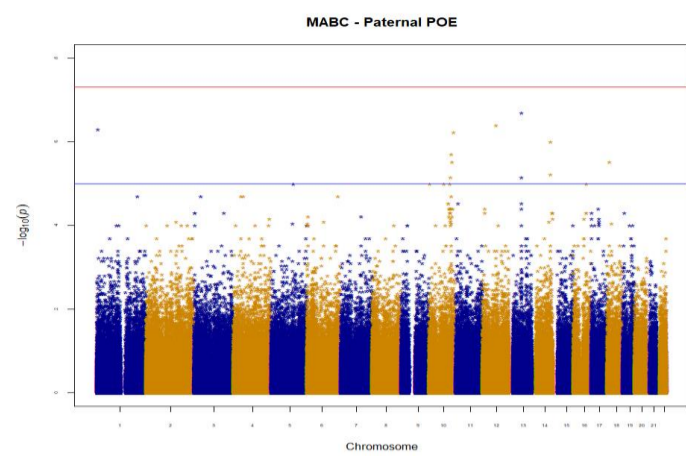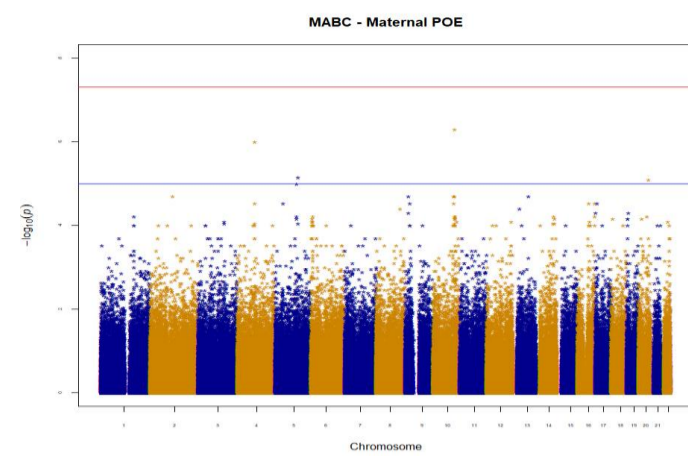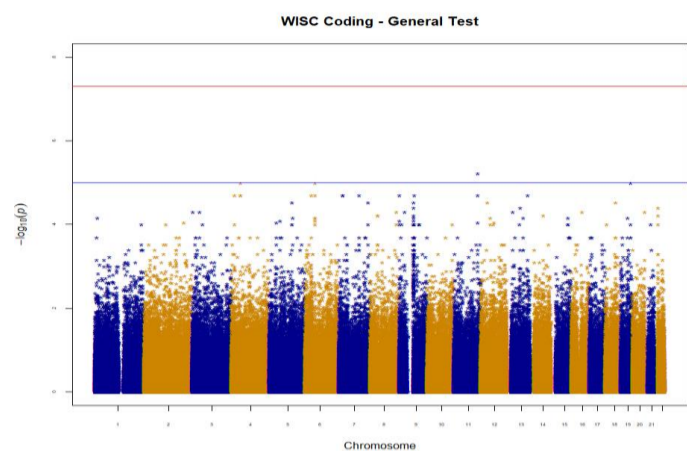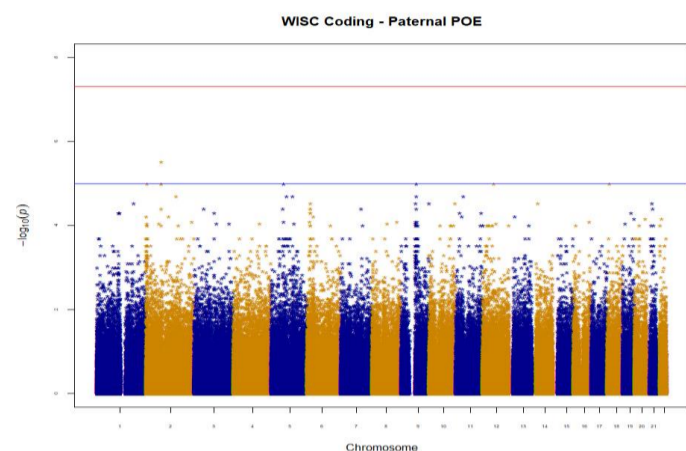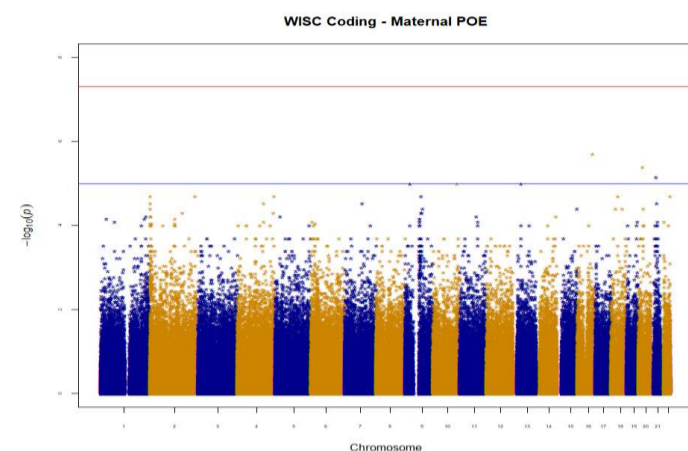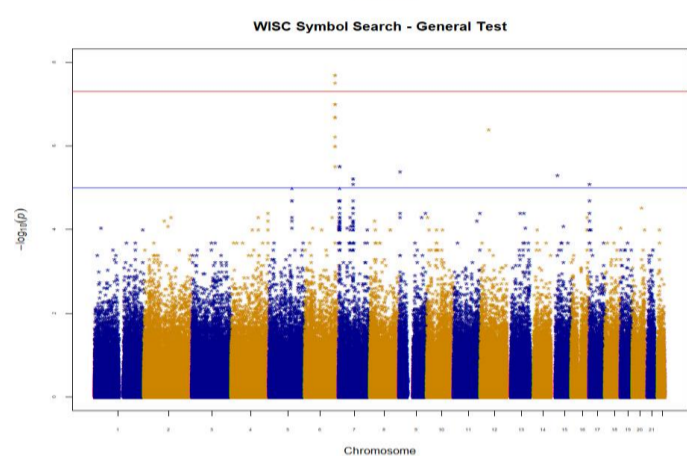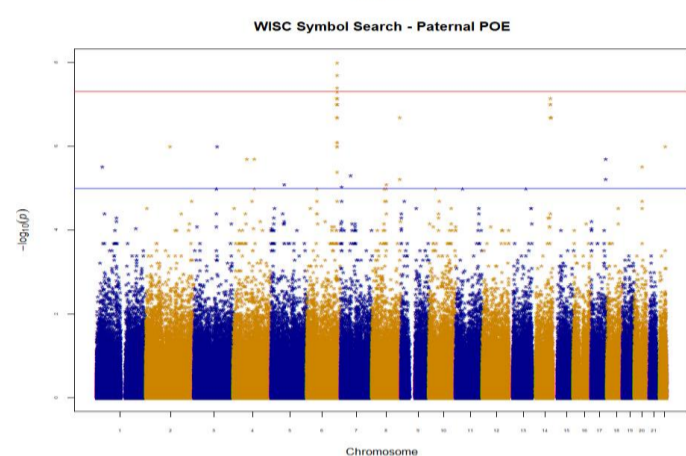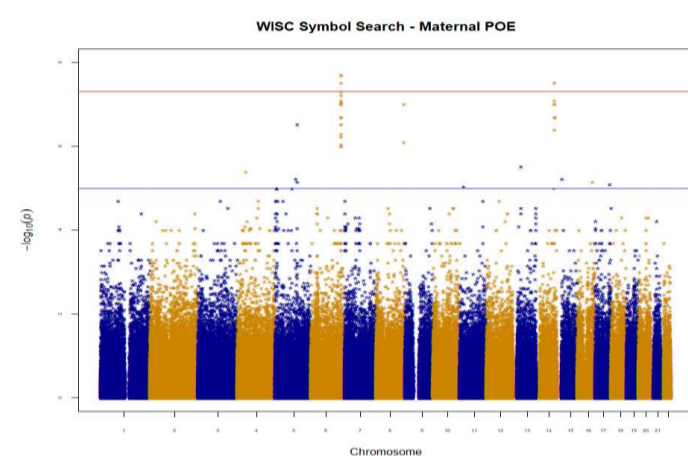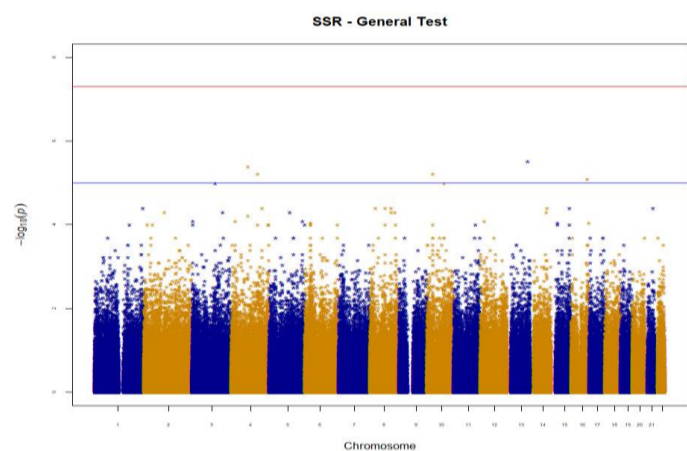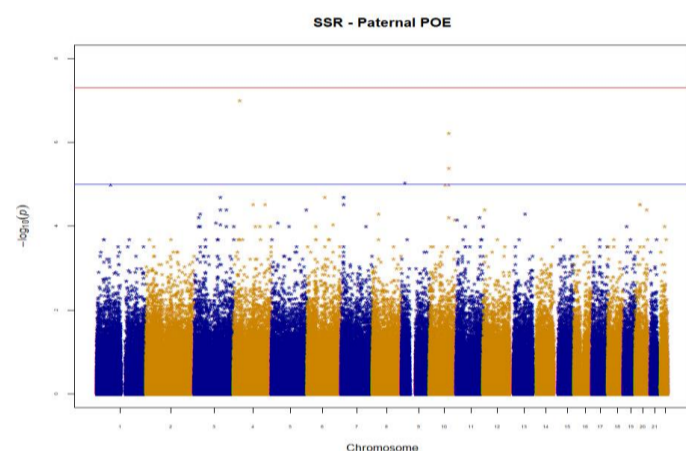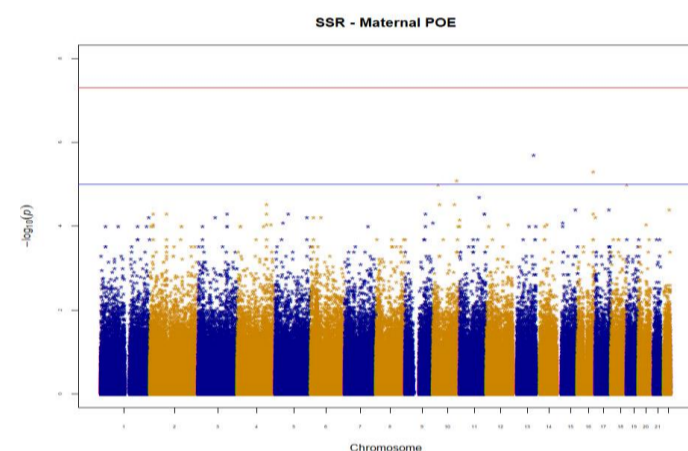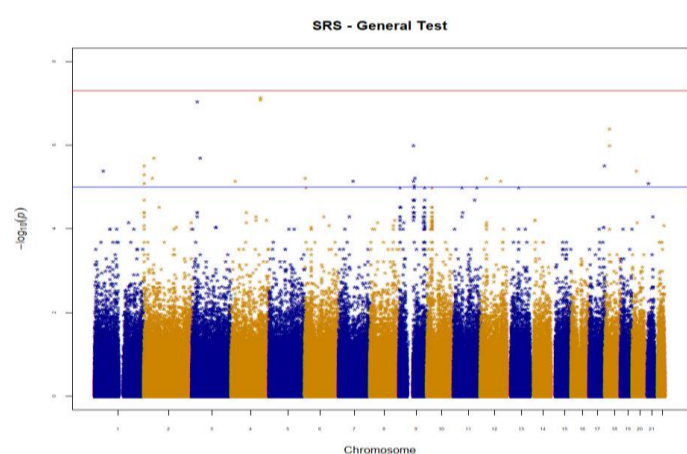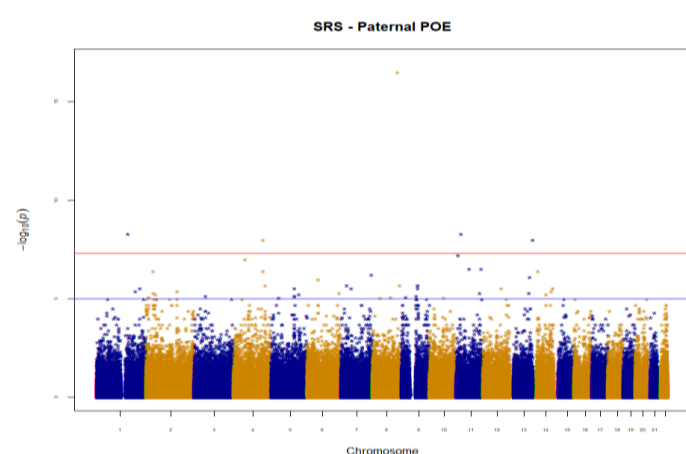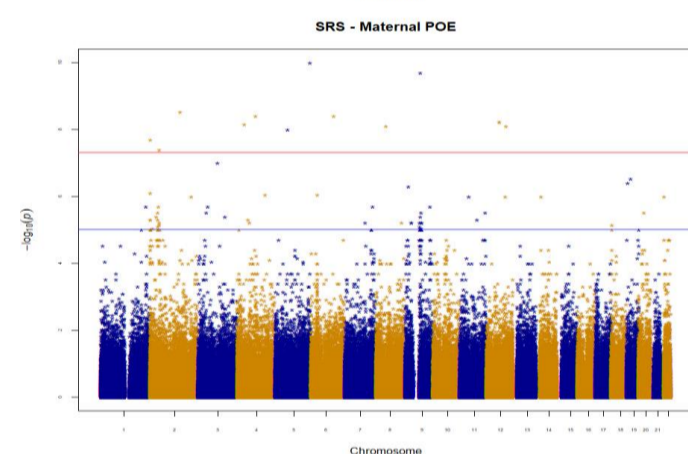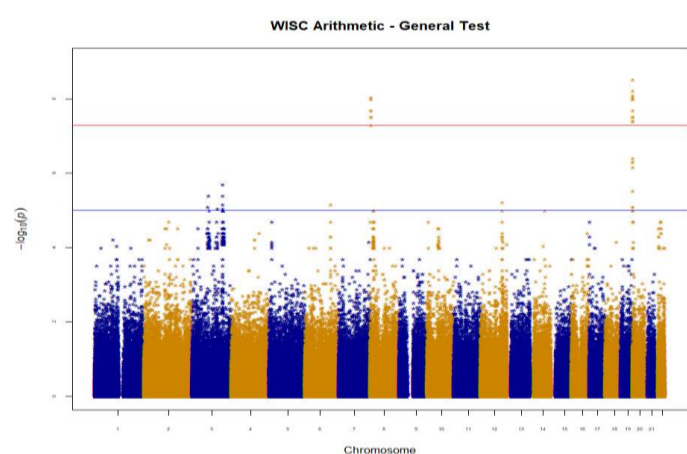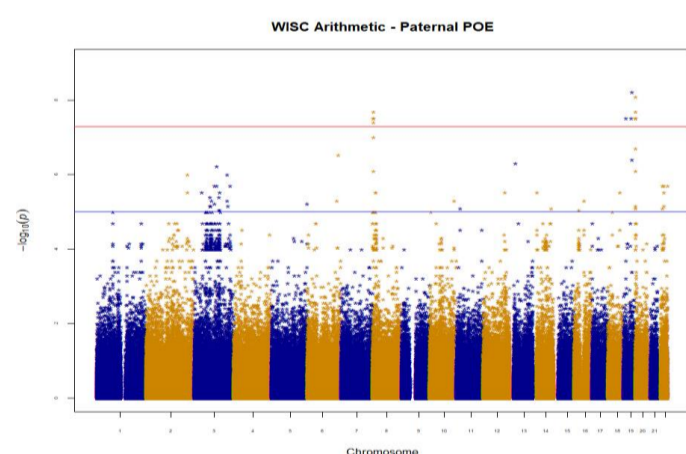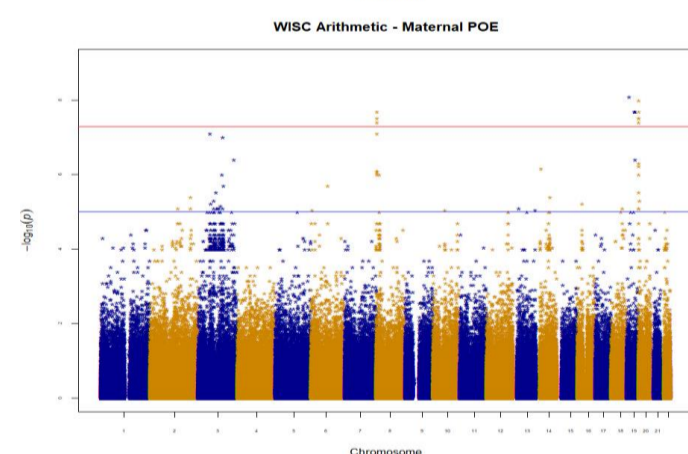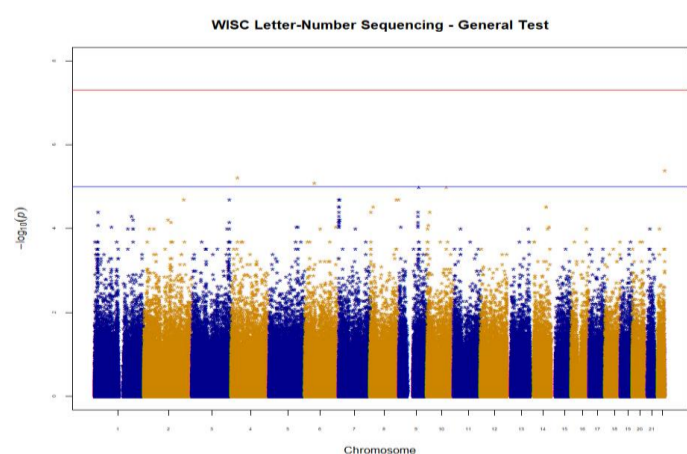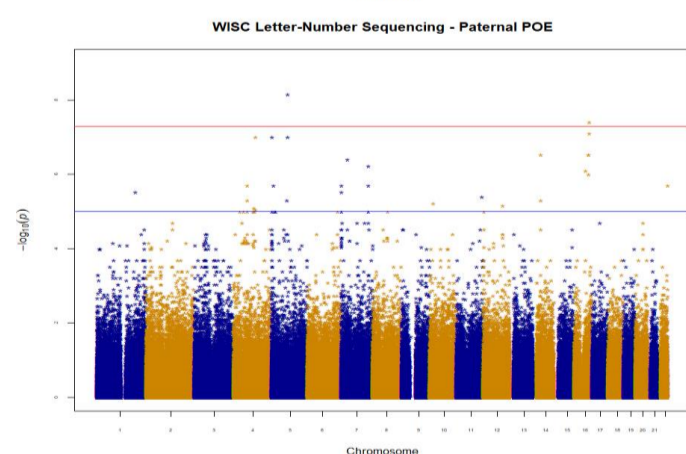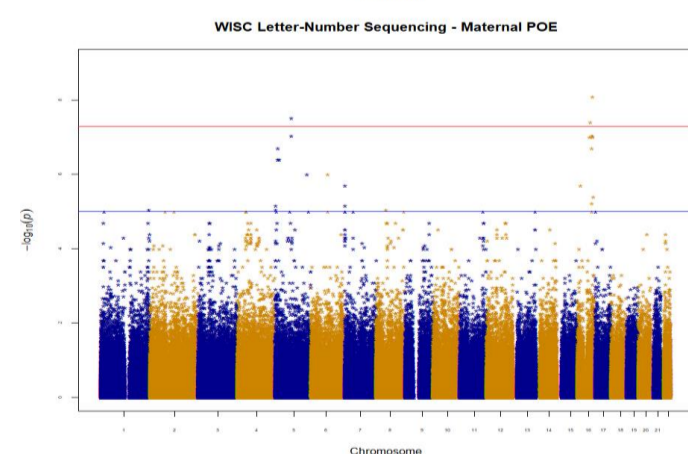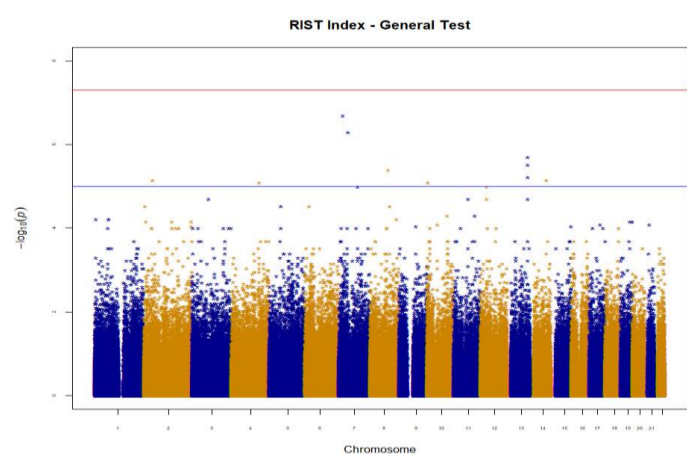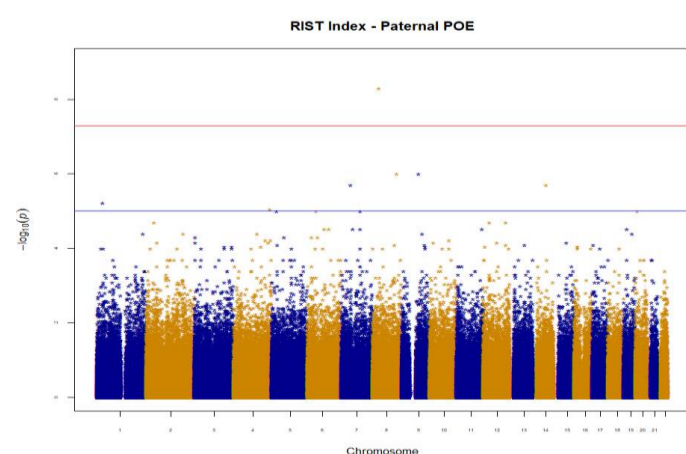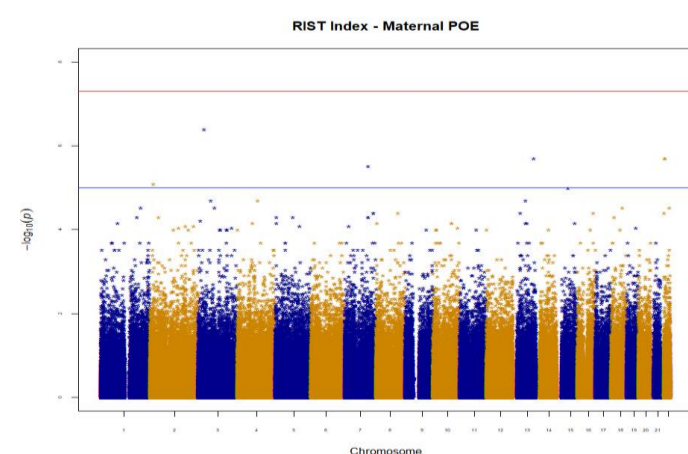

Supplement: Supplementary file 2 — Additional file 2: Figure S1. Manhattan plots for all 24 GWASs. [file 12993_2022_198_MOESM2_ESM.pdf]

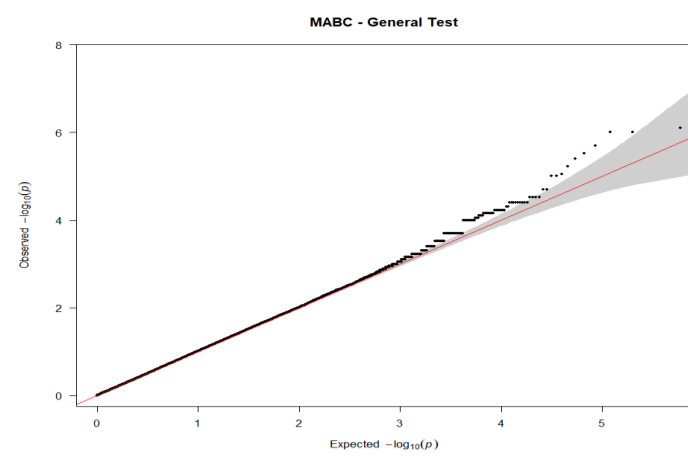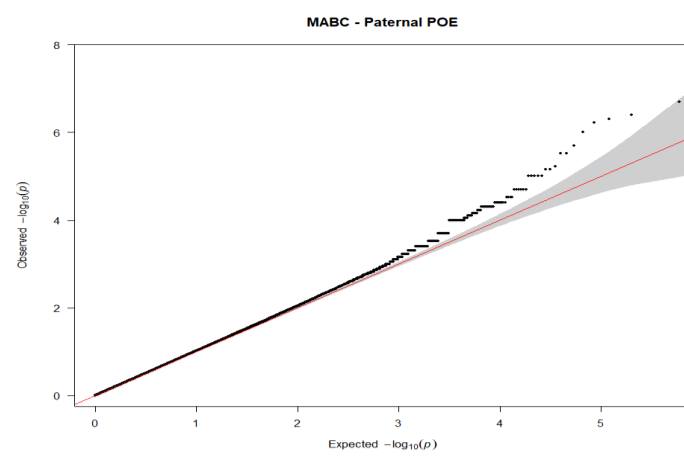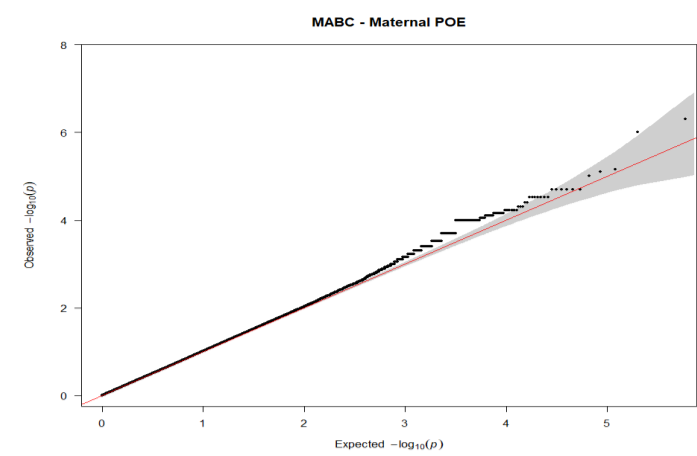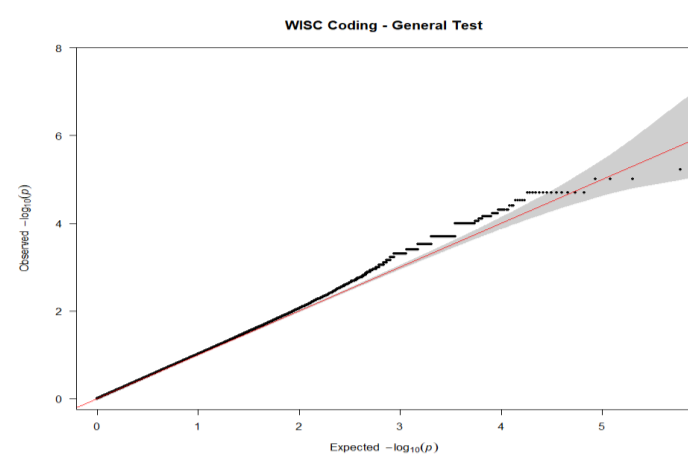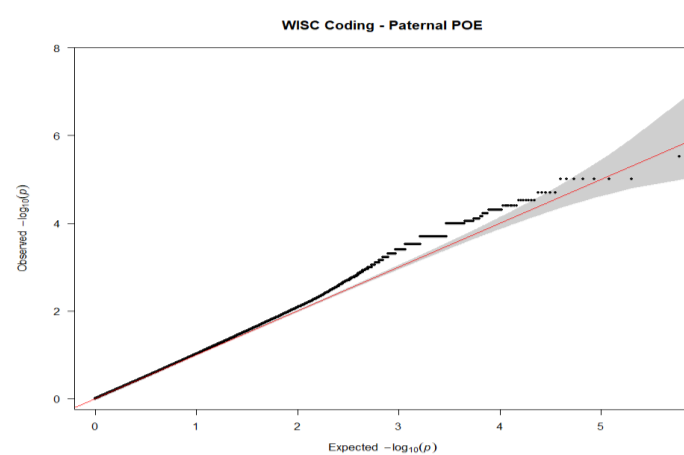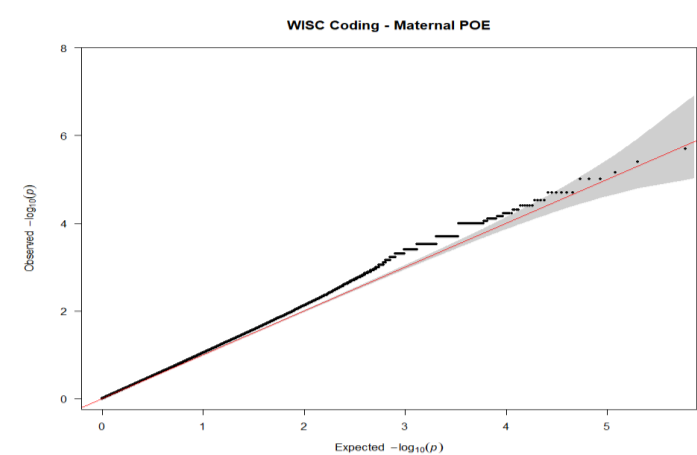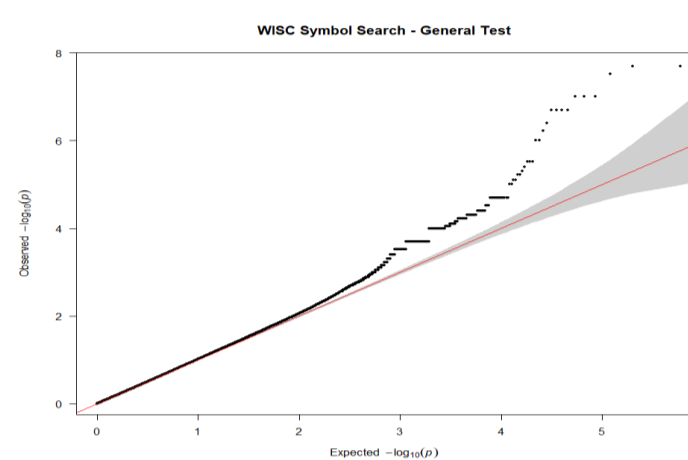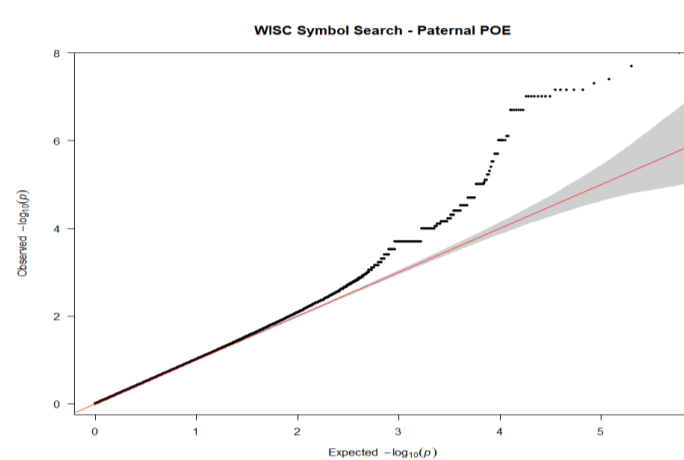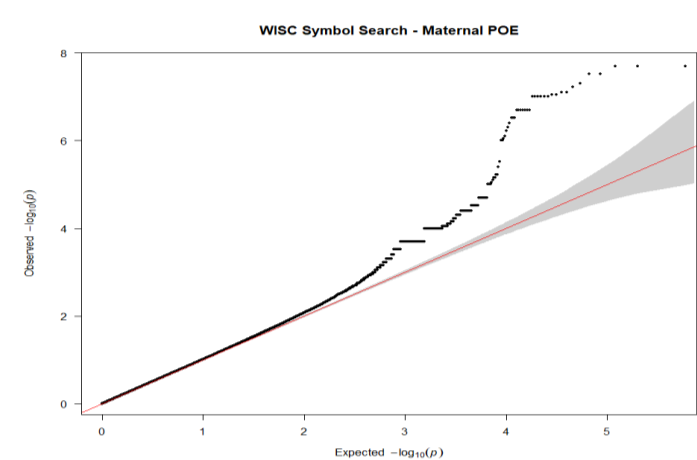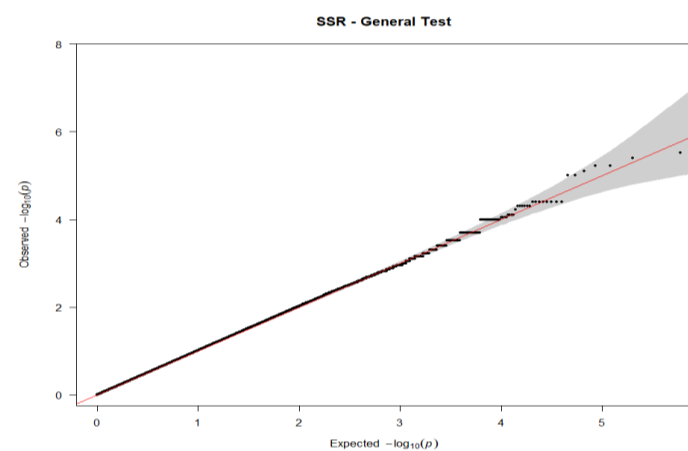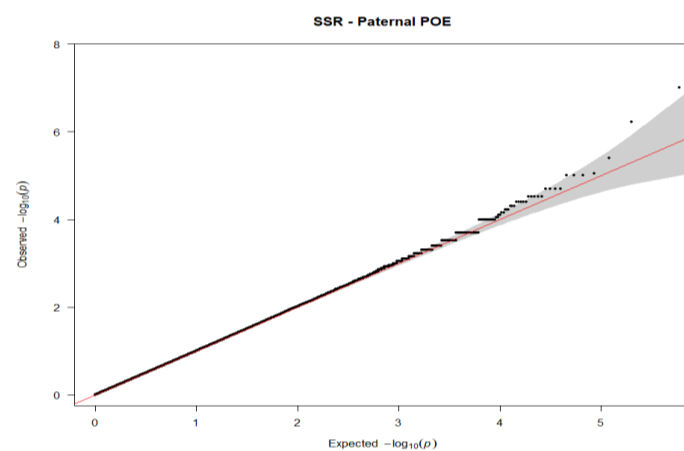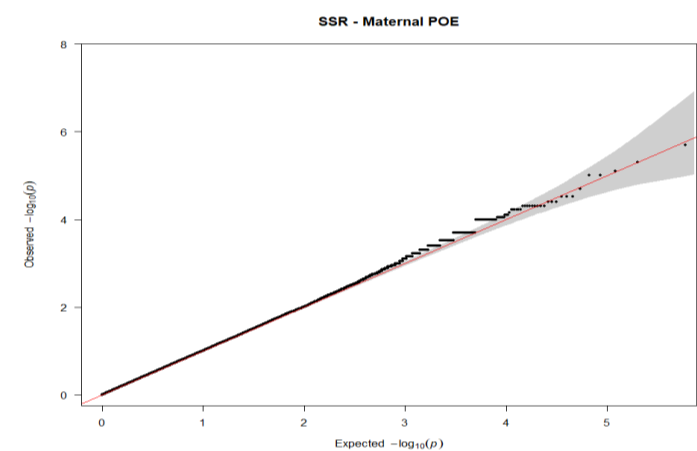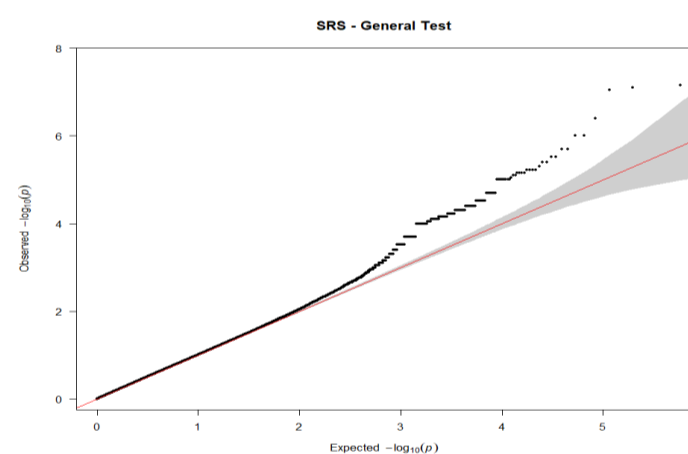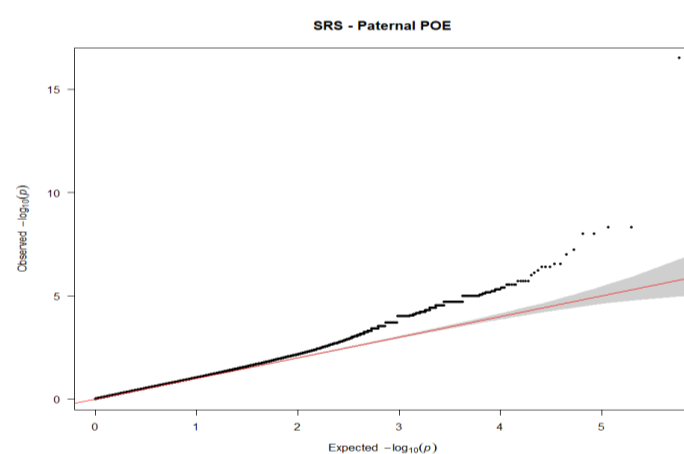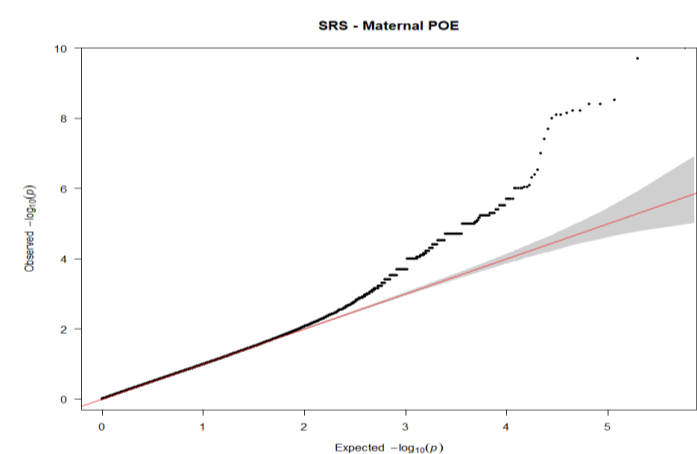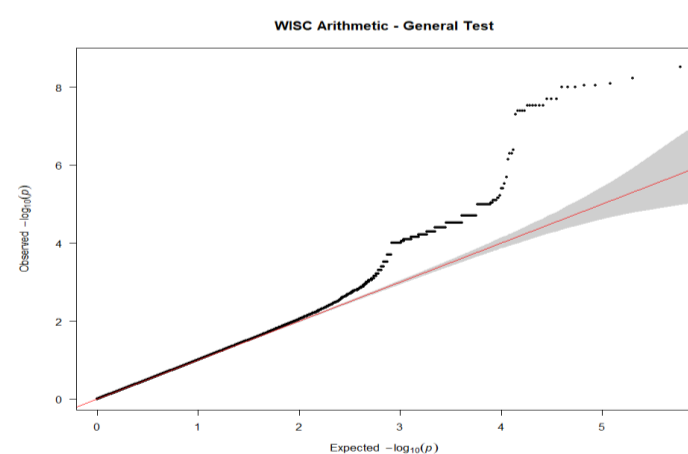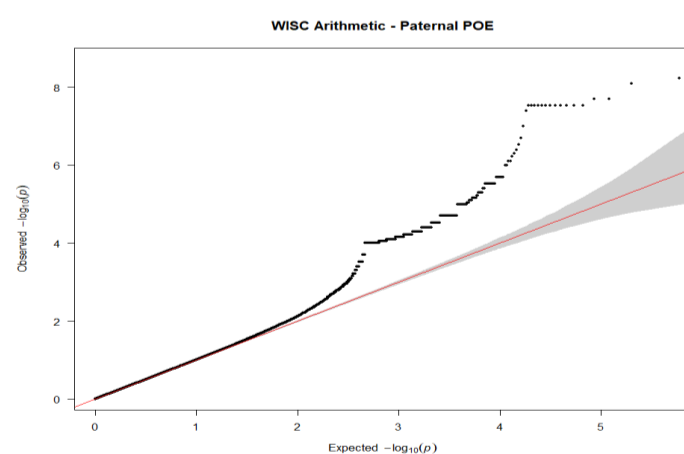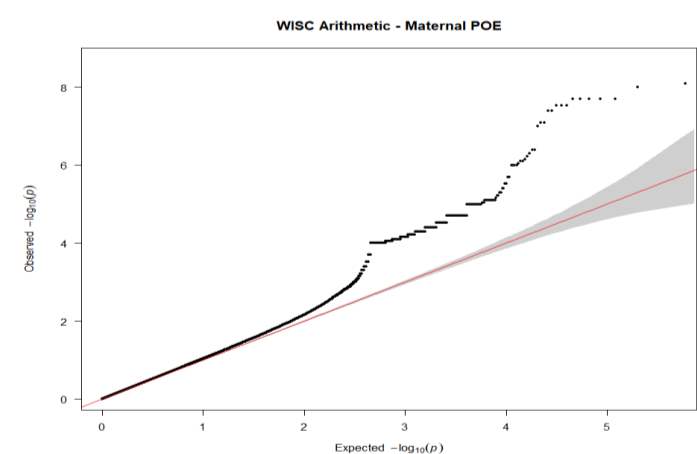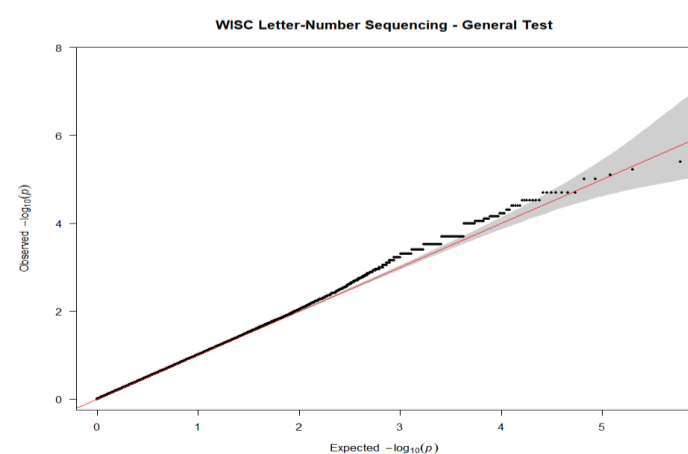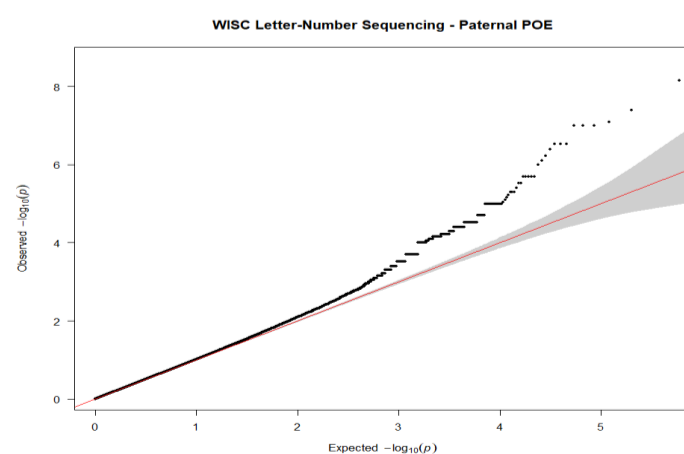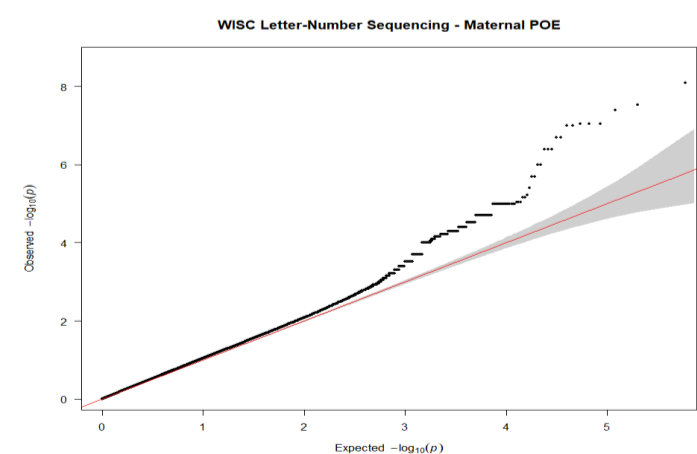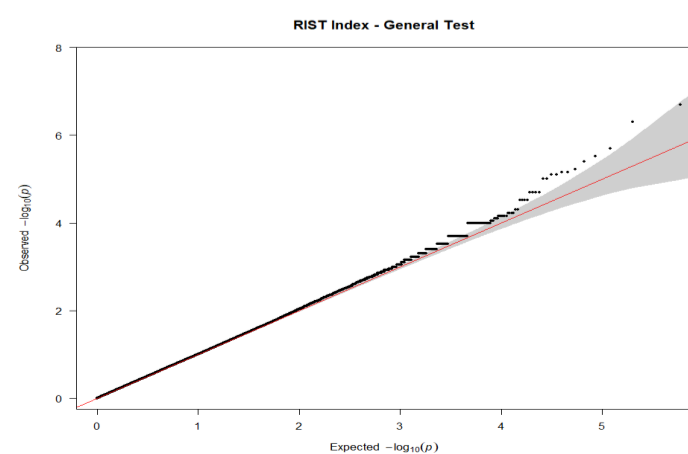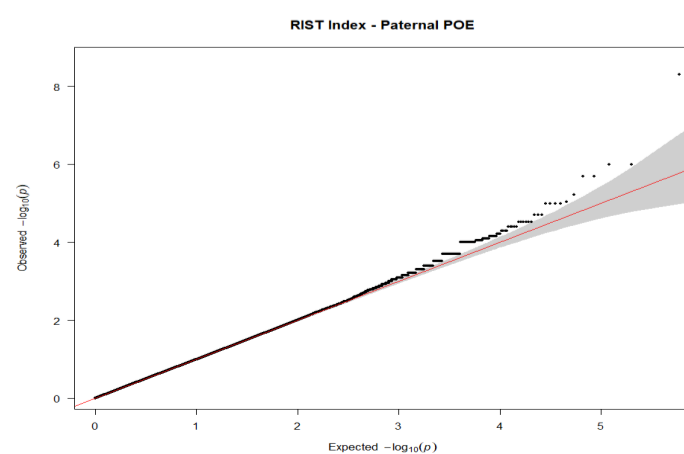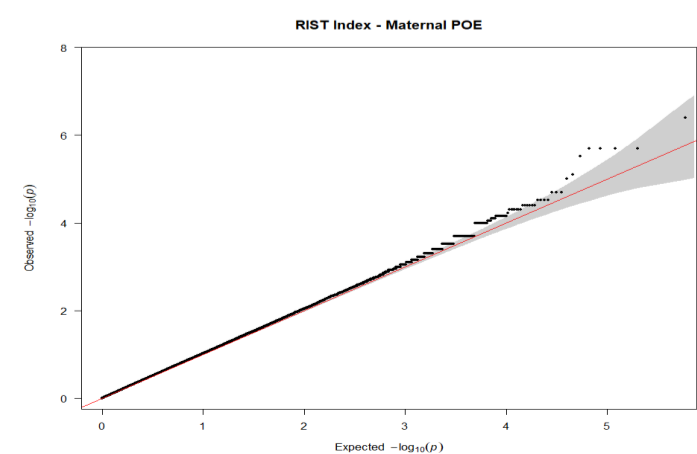

Supplement: Supplementary file 3 — Additional file 3: Figure S2. QQ plots for all 24 GWASs. [file 12993_2022_198_MOESM3_ESM.pdf]
