# Supplementary material for: A family-based study of genetic and epigenetic effects across multiple neurocognitive, motor, social-cognitive and social-behavioral functions
Source: Behav Brain Funct. 2022 Dec 1;18:14. doi: 10.1186/s12993-022-00198-0 (PMC9714039; doi:10.1186/s12993-022-00198-0)
Supplement: Supplementary file 4 — Additional file 4: Table S1. Direct associations from VarElect for genes in Table 1. [file 12993_2022_198_MOESM4_ESM.pdf]

Table S1. Direct associations from VarElect for genes in Table 1.

Symbol	Description	Matched Terms	Matched Terms Count	Score	Average Disease Causing Likelihood
CSMD1	CUB And Sushi Multiple Domains 1	autism, "working memory", behavior, communication, intelligence, schizophrenia, "bipolar disorder"	7	**23.89**	61.87%
SYNE1	Spectrin Repeat Containing Nuclear Envelope Protein 1	autism, "working memory", behavior, communication, intelligence, "processing speed", schizophrenia, "bipolar disorder"	8	**16.37**	46.49%
CPLX2	Complexin 2	"working memory", behavior, communication, intelligence, schizophrenia, "bipolar disorder"	6	**11.31**	72.31%
ZSWIM6	Zinc Finger SWIM-Type Containing 6	autism, behavior, communication, intelligence, schizophrenia, "bipolar disorder"	6	**11.16**	Not determined
CACNB4	Calcium Voltage-Gated Channel Auxiliary Subunit Beta 4	autism, behavior, communication, intelligence, schizophrenia, "bipolar disorder"	6	**6.13**	85.30%
ANKS1B	Ankyrin Repeat And Sterile Alpha Motif Domain Containing 1B	autism, "working memory", behavior, communication, intelligence, "processing speed", schizophrenia, "bipolar disorder"	8	**4.65**	68.18%
PRKCE	Protein Kinase C Epsilon	behavior, communication, intelligence, "bipolar disorder"	4	**2.96**	71.02%
SIRPA	Signal Regulatory Protein Alpha	"working memory", behavior, communication, schizophrenia	4	**1.63**	12.53%
KIF13B	Kinesin Family Member 13B	behavior, communication, schizophrenia	3	**0.71**	68.84%
TGM3	Transglutaminase 3	behavior, communication	2	**0.49**	15.96%
FRK	Fyn Related Src Family Tyrosine Kinase	behavior, communication, schizophrenia	3	**0.46**	20.75%
ATP11A	ATPase Phospholipid Transporting 11A	communication, schizophrenia, "bipolar disorder"	3	**0.37**	69.18%
CEMIP2 (TMEM2)	Cell Migration Inducing Hyaluronidase 2	communication	1	**0.20**	47.18%
CLXN (EFCAB1)	Calaxin	communication	1	**0.02**	69.09%

Definitions from the VarElect website:

Score: This score is an indication of the strength of the connection between the gene and the queried phenotype/s. Scores usually range from 1 [to] 200. The main purpose of the score is to enable ranking and prioritizing the list of queried genes by relevance to the queried phenotype/s. Scores from different runs cannot and should not be compared, since relevance scores produced for each run are relative within the run and not absolute.

Average disease causing likelihood: The Disease Causing Likelihood column reflects the principle that a variant in a gene with high mutation intolerance is more likely to be disease causing. RVIS is residual variation intolerance score (Petrovski et al.) and GDI is Gene Damage Index (Itan et al.). Disease- causing likelihoods are 100% minus RVIS percentile or 100% minus GDI percentile, with the average of both shown numerically.
